# Characterization and spatiotemporal variations of fluorescent dissolved organic matter in leachate from old landfill-derived incineration residues and incombustible waste

**DOI:** 10.1371/journal.pone.0304188

**Published:** 2024-06-26

**Authors:** Thi Ngoc Nguyen, Taketoshi Kusakabe, Masaki Takaoka

**Affiliations:** Department of Environmental Engineering, Graduate School of Engineering, Kyoto University, C-cluster, Katsura Campus, Kyoto, Japan; Linköping University: Linkopings universitet, SWEDEN

## Abstract

Dissolved organic matter (DOM) influences the bioavailability and behavior of trace metals and other pollutants in landfill leachate. This research characterized fluorescent dissolved organic matter (FDOM) in leachate from an old landfill in Japan during a 13-month investigation. We employed excitation–emission matrix (EEM) fluorescence spectroscopy with parallel factor analysis (PARAFAC) to deconvolute the FDOM complex mixture into three fluorophores: microbial humic-like (C1), terrestrial humic-like (C2), and tryptophan-like fluorophores (C3). These FDOM components were compared with findings from other studies of leachate in landfills with different waste compositions. The correlations among EEM-PARAFAC components, dissolved organic carbon (DOC) concentration, and ultraviolet–visible and fluorescence indices were evaluated. The FDOM in leachate varied spatially among old and extended leachate collected in the landfill and leachate treatment facility. The FDOM changed temporally and decreased markedly in August 2019, November 2019, and April 2020. The strong positive correlation between HIX and %C2 (*r* = 0.87, *ρ* = 0.91, *p* < 0.001)) implies that HIX may indicate the relative contribution of terrestrial humic-like components in landfill leachate. The F_max_ of C1, C2, and C3 and the DOC concentration showed strong correlations among each other (*r* > 0.72, *ρ* > 0.78, *p* < 0.001) and positive correlations with leachate level (*r* > 0.41, *p* < 0.001), suggesting the importance of hydrological effects and leachate pump operation on FDOM.

## Introduction

In European countries and Japan, incineration has become the prevailing intermediate treatment for MSW since the 1970s [1–3]. Incineration residues include bottom ash and fly ash, which are ultimately disposed of in sanitary landfills [2]. Dissolved organic matter (DOM) in landfill leachate is a complex mixture, which can originate from incineration residues, incombustible waste combined with microbial activities in landfills, and soil covering landfills [2, 4–7]. However, recognizing specific properties of DOM and tracing its origins in landfill leachate is challenging due to significant variations in the composition of solid waste, which serves as a source of DOM at landfill sites [4]. The characteristics of DOM are considered to exhibit variations among sampling locations, potentially influencing the bioavailability, toxicity, and migration of trace metals and other pollutants to the surrounding environment [8, 9]. He et al. [10] emphasized the importance of treating DOM in landfill leachate for safe disposal. Old landfill sites will ultimately be abolished once decomposition has stabilized after a monitoring period [11]. Therefore, there is a need for a practical understanding of the variations in DOM within landfills, to assist in decisions to landfill abolition.

Excitation–emission matrix (EEM) fluorescence spectroscopy has been used widely to characterize and monitor fluorescent dissolved organic matter (FDOM) in various environments, including marine water, freshwater, and wastewater [9, 12–15], owing to its relative simplicity, high sensitivity and specificity, and rapid analysis. Nonetheless, the EEM spectra of DOM cannot be easily interpreted due to overlaps in the spectra of different fluorophores [16]. Various methods have been developed to characterize the fluorescence datasets of organic matter, such as the peak-picking technique, fluorescence regional integration (FRI), parallel factor analysis (PARAFAC), principal component analysis (PCA), and self-organizing maps (SOMs) [14, 17, 18]. Among these methods, PARAFAC is a popular multivariate modeling technique that deconvolutes the EEM spectra of FDOM into distinct fluorescent components to quantify, relatively, each component [19]. Although DOM characterization using EEM-PARAFAC has been widely reported in various environments [9, 12–15], including landfill leachate, most studies have investigated direct landfills containing unincinerated MSW, or a combination of MSW and incineration residues [9, 17, 20]. In previous studies, FDOM was deconvoluted into 2 to 6 distinct components, emphasizing the differences in the number of PARAFAC components and their assignments among various landfills. In traditional Chinese landfills, including unburned waste, four EEM-PARAFAC-derived components were assigned, comprising one fulvic-like substance, two humic-like substances, and one protein-like compound [17]. In another Chinese sanitary landfill site, four humic-like and two protein-like components were identified by PARAFAC [9], while leachate FDOM was deconvoluted into only one humic-like and one tryptophan-like component in a study from a stabilized Korean landfill [21]. By contrast, the DOM in the leachate of landfills containing only incineration residues and incombustible waste remains poorly understood. Most investigations on spatiotemporal variations of FDOM have mainly focused on rivers [22–24] and lakes [25, 26] rather than landfill leachate. Moreover, reports on DOM in landfills have focused mainly on improving leachate treatment [14, 27]. Only Lu et al. [28] have reported on the organic matter in leachate at multiple sites within a single landfill. Overall, studies on the spatial distributions have been conducted in relatively new landfills operating for only 3–15 years; the behavior of organic matter in older landfills (e.g., operating for 40 years) has not been reported.

In addition, the humification index (HIX) and specific ultraviolet absorbance at 254 nm (SUVA_254_) have been commonly used as indicators of the humic substance content, and the humification degree and aromaticity of DOM [29, 30]. SUVA_254_ was calculated dividing the absorption coefficient at 254 nm (cm^–1^) by DOC concentration (mg C/L), and then multiplying by 100 (cm/m) [29], while HIX was calculated by dividing the area under the Em spectra 435–480 nm by the peak area 300–345 nm + 435–480 nm, at Ex 254 nm [30]. Numerous studies have shown positive correlations between the HIX, SUVA_254_, and humic-like components derived from EEM-PARAFAC [31, 32]. Understanding humic substances or humus content and humification degree of organic matter is crucial for characterizing the landfill waste stability, the decomposition process and selecting suitable leachate treatment [33, 34]. However, He et al. [33] suggested that several humification indices showed no correlation with the F_max_ of humic-like substances and were not suitable for characterizing humification. In the present study, we assessed whether the indicators HIX and SUVA_254_ correlated with any EEM-PARAFAC components and fluorescence parameters in landfill leachate.

In light of these gaps in the literature, our study aims to address the following research questions: What are the specific characteristics and variations of FDOM in the leachate of an old municipal landfill containing incineration residues and incombustible waste, which had been landfilled for 25–44 years? How do these characteristics compare with existing studies on landfill leachate? What are the relationships among fluorescence components, spectroscopic parameters, and dissolved organic carbon (DOC) concentration?

To address the aforementioned research questions, PARAFAC was applied to deconvolute the fluorescence signals into the underlying individual fluorescent components. Subsequently, these components were compared with findings from other studies of landfill leachate. The characterization and variations of EEM-PARAFAC components were evaluated alongside results derived from the FRI method, serving as a comparative method. Furthermore, the spatial and temporal variations in FDOM in leachate across the study site were investigated, and leachate DOM was assessed before and after treatment. Correlation and multilinear regression analysis were employed to understand the relationships among fluorescence components, spectroscopic parameters, and DOC concentration. Finally, the utility of indicators such as HIX and SUVA_254_, based on their correlation with humic-like components derived from EEM-PARAFAC, was evaluated and discussed.

## Materials and methods

### Landfill site and sample collection

**Fig 1:**
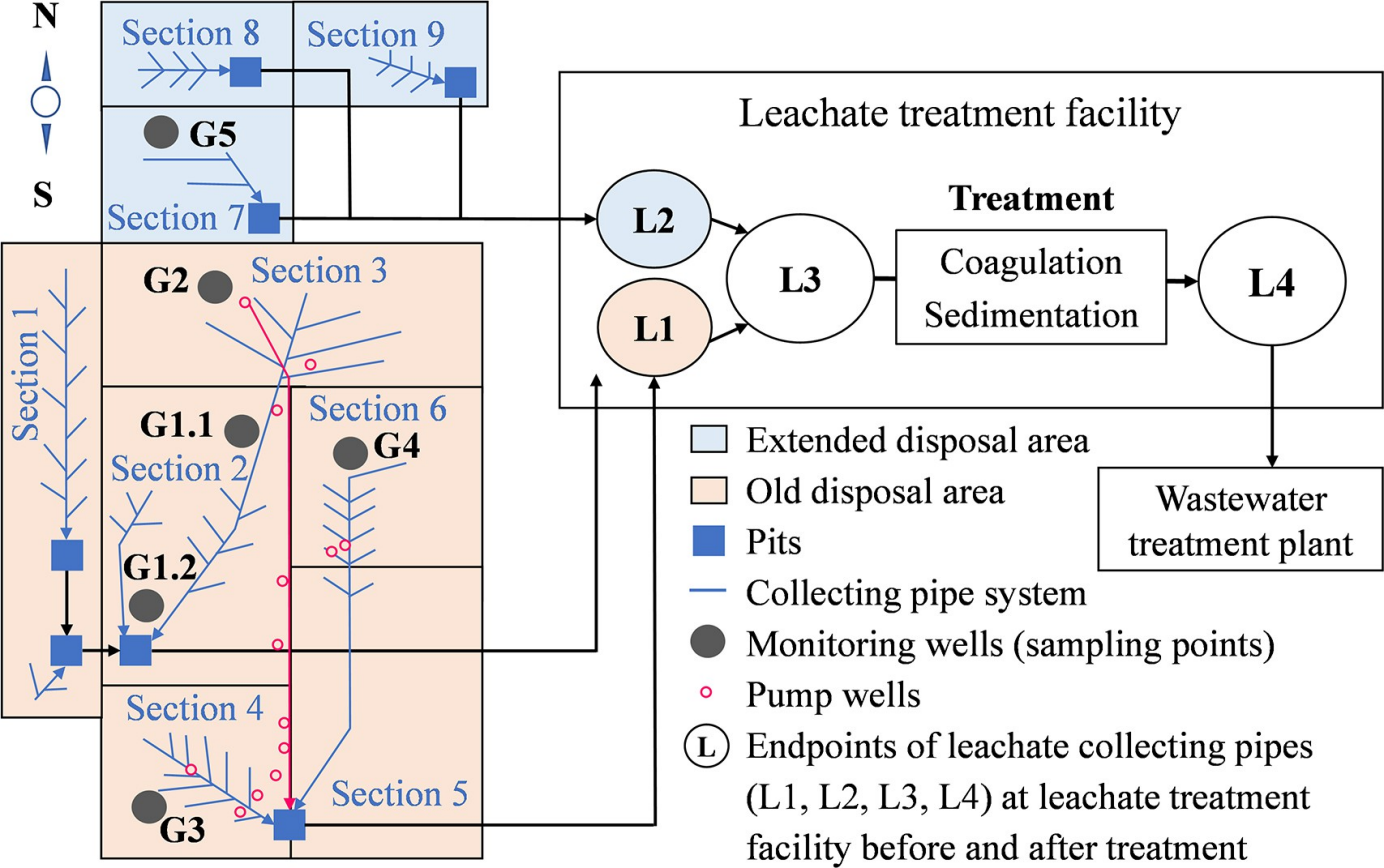
Schematic overview of the landfill site and leachate-collecting pipe system.

The landfill site, located in a city in Japan, contained approximately 75% of incineration residues (bottom ash and fly ash) and 25% of incombustible waste consisting mainly of concrete, asphalt, glass, and ceramics [3]. Fig 1 presents a schematic overview of the landfill, leachate-collecting pipe system, and monitoring wells. In the landfill, we installed sampling devices containing a smart groundwater purging pump (Daiki Rika Kogyo, Saitama, Japan) at six monitoring wells to monitor leachate water quality. Over 13 months, from June 2019 to June 2020, 130 leachate samples were collected monthly at 10 sampling points. Polypropylene (PP) plastic bottles (Sanplatec, Osaka, Japan) were thoroughly cleaned and rinsed with Milli-Q water, followed by three additional rinses with the samples, prior to their use for sample collection. Six samples were collected in the monitoring wells (G1.1, G1.2, G2, G3, G4, and G5) located in five sections that covered most of the landfill area. The landfill operation periods varied among sections: G1.1 and G1.2 (1976–1985), G2 (1977–1986), G3 (1985–1989), and G4 (1987–1993) from the old disposal area, and G5 (1994–1995) from the extended disposal area. Four additional leachate samples (L1, L2, L3, and L4) were collected at the leachate treatment facility. L1 was leachate collected from a pit located at the end of sections 4 and 5 in the old landfill area. L2 consisted of leachate collected from the extended landfill area, including G5. L3 comprised a mix of the untreated leachate downstream of L1 and L2. Finally, L4 was the leachate post-treatment. All samples were transported in iceboxes to the laboratory as quickly as possible.

### DOC analysis

To prevent contamination from organic matter, pre-combusted glass fiber filters (Whatman GF/B, 1 μm; Whatman, Maidstone, UK) were used to separate solid and dissolved matter [35]. The DOC concentration was measured as non-purgeable organic carbon using the TOC-V_CSH_ analyzer (Shimadzu, Kyoto, Japan). The detection limit (LOD) and quantification limit (LOQ) were 0.03 mg C/L and 0.1 mg C/L, respectively. The DOC concentration of each leachate sample was determined by averaging measurements conducted at least twice until the coefficient of variation (CV) was < 2%.

### Spectroscopic analysis

Ultraviolet–visible absorbance scans were performed from 600 to 250 nm using a 1-cm quartz cuvette and UV-1280 spectrophotometer (Shimadzu) with a wavelength step size of 1 nm before fluorescence measurement to correct for inner filter effects (IFEs). The inner filter correction procedure is described in S1 Text of S1 File. Samples were allowed to stand at room temperature (20°C) before the spectroscopic measurements. Fluorescence measurements were conducted using the RF-5301 PC spectrofluorophotometer (Shimadzu) and a quartz cuvette. The scanning ranges were 250–450 nm (5-nm increments) and 250–600 nm (5-nm increments) for the excitation and emission wavelengths, respectively, with a slit width of 5 nm for both excitation and emission monochromators and 150 W xenon lamp operated in the very fast wavelength speed mode. All samples were acidified to pH 2.0 ± 0.1 using HCl for both DOC and spectroscopic analysis. The fluorescence intensities were normalized to Raman units using the signal intensity of the Raman peak of Milli-Q (18.2 MΩ) water, which was also adjusted to pH 2.0 ± 0.1 using HCl at excitation/emission wavelengths (Ex/Em) of 350/350–450 nm. The relative precision of fluorescence intensity measurements was <2% based on repeated measurements.

### Data analyses

#### PARAFAC

The EEMs were modelled by PARAFAC [12, 36] in the MATLAB software 2018b environment (MathWorks, Natick, MA, USA). The principle and formula of PARAFAC are presented in S2 Text of S1 File. PARAFAC was conducted on 126 samples collected at 10 sites in the landfill and wastewater treatment facility from June 2019 to June 2020, using the drEEM toolbox (version 0.6.0) based on the tutorial of Murphy et al. [12]. Four EEMs spectra were removed from the initial 130 EEMs spectra because one sample had an abnormal fluorescence intensity, and three samples had fluorescence intensities exceeding the upper maximum limit of 1000 AU [37].

In the procedure for PARAFAC analysis of EEMs (S1 Fig in S1 File), we first collected and pre-processed the data. Pre-processing consisted of EEM assembly, IFE correction, Raman normalization, scatter removal, interpolation, and signal normalization. Next, preliminary models of different components were run, outliers were removed, and model fits were evaluated. The FDOM components were identified and validated using jack-knifing, residual analysis, the core consistency diagnostic, and split-half validation [12, 38, 39]. All three validation approaches (residuals and loadings analysis, core consistency, and split-half analysis) indicated that the three-component model was optimal. Finally, the results were obtained as the maximum fluorescence intensity, *x*_*ijk*_, and excitation and emission loadings for each sample. The detailed procedure and results are shown in S3 and S4 Texts, S1 Table, S2–S9 Figs of S1 File.

#### Correlation analysis and multilinear regression analysis

The samples collected at 10 sampling sites from June 2019 to June 2020 were used for correlation analysis (sample size, *n* = 126). The maximum fluorescence intensity (F_max_) and the relative percentage of each component derived from EEM-PARAFAC (%C1, %C2, %C3), fluorescent peak ratios (C:M, C:T, C:A, A:T, M:T) [40] were used as the input data for the correlation analysis. Several optical indices were added, including the specific ultraviolet absorbance at 254 nm (SUVA_254_) [29], fluorescence index (FI) [41, 42], humification index (HIX) [30], biological index (BIX) [43], and DOC concentration. The detailed descriptions of these optical indices are shown in S2 Table of S1 File. Both Pearson’s r values and Spearman’s rho were evaluated in correlation analysis because most of the data were not normally distributed [44]. Multilinear regression analysis was used to predict the value of a dependent variable DOC concentration based on the values of independent or predictor variables (F_max_ values of f C1, C2 and C3). These analyses were conducted on R version 4.3.3.

#### Fluorescence regional integration (FRI)

The FRI method divides the EEM spectra into two regions and characterizes FDOM by calculating the volume of each region [37, 45]. A detailed method description is presented in S5 Text of S1 File. The characterizations and variations of FDOM derived from the EEM-PARAFAC method were compared with those obtained from the FRI method.

All experimental and computational analyses described in this manuscript were conducted at Katsura Campus, Kyoto University in Japan.

## Results and discussion

### EEM-PARAFAC model and comparison with other studies

**Fig 2:**
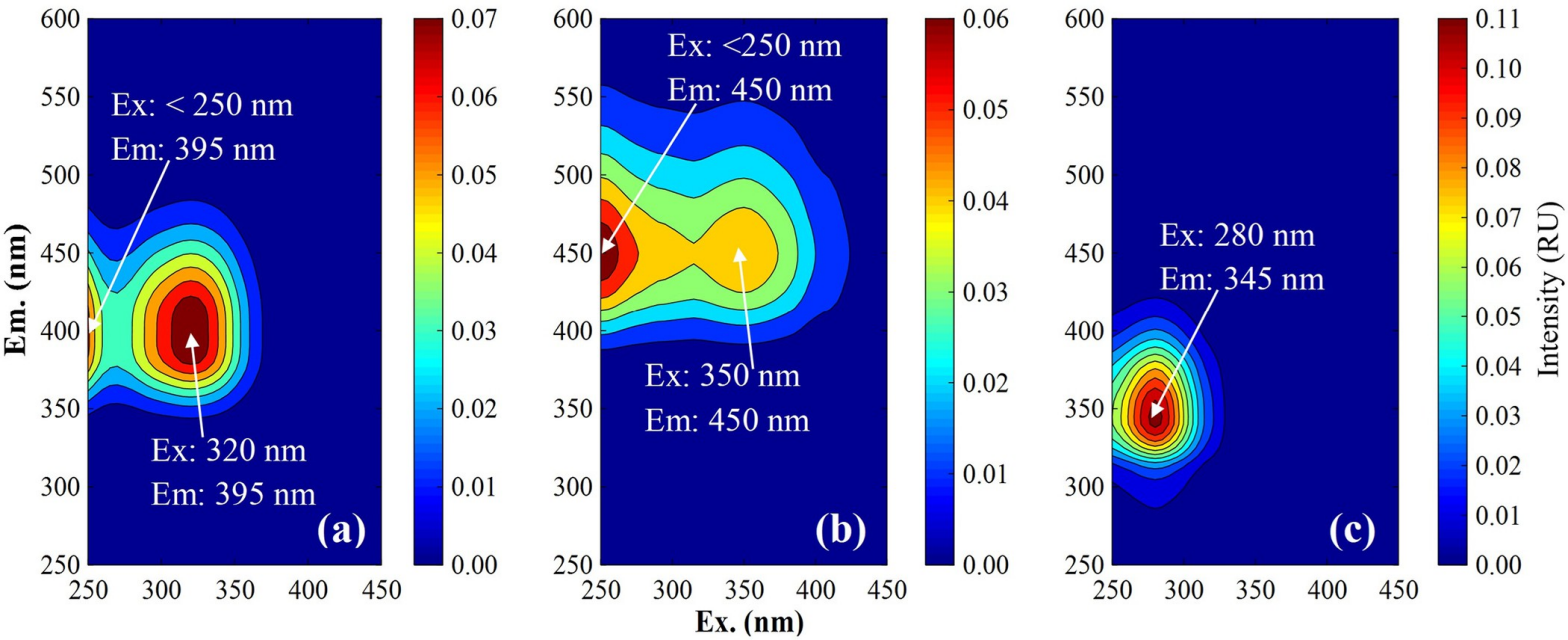
Fluorescence components identified by EEM-PARAFAC analysis. **Three** (a) Component C1, (b) Component C2, (c) Component C3.

Fig 2 illustrates the contours of the three fluorescent components deconvoluted by PARAFAC: Component 1 (C1) at Ex/Em < 250, 320/395 nm, Component 2 (C2) at Ex/Em < 250, 350/450 nm, and Component 3 (C3) at Ex/Em 280/345 nm. The peak positions of these components were compared with those in studies of FDOM spectra in other landfill leachate samples in China, Korea, and Belgium (Table 1). The waste compositions of some of these landfills consisted of MSW, sewage sludge, solidified sludge, domestic waste, and construction waste; however, the previous studies typically did not provide detailed information on the type of waste or type of landfill site. In previous research, leachate samples were collected in the landfill or during different stages of MSW management. By contrast, the waste composition of the landfill in the present study comprised mainly incineration residues and incombustible waste. Research on FDOM in the leachate of landfills containing primarily incinerated MSW remains poorly understood. A component similar to C1 in this research was reported as a fulvic acid-like substance related to aromatic and aliphatic groups of leachate-derived DOM [17, 20, 27] or microbial humic-like components derived from microbial activity in landfills [21, 46, 47]. Oloibiri et al. [14] assigned compound C1 as humic- and fulvic-like components, with the first peak at 250/425 nm representing humic substances and the second peak at 320/425 nm representing fulvic-like compounds in DOM. However, the peak emission wavelength of C1 in this research (at 395 nm) was shorter than the values from other studies of leachate FDOM (400–425 nm). Only Zhang et al. [47] observed a similar peak position as C1 for the emission wavelength (390 nm); however, the excitation wavelength differed (280, 330 nm) compared with our findings (< 250, 320 nm). Similar component of C2 in this study were assigned as humic-like component [9, 17, 20] or terrigenous humic-like component [47] or terrestrial humic-like component [21, 48]. Similar component of C3 were assigned as protein-like component [9, 17] or tryptophan-like component [20, 27, 46].

**Table 1:** PARAFAC–derived components in this research compared with previous studies.

**Component**	**Peak assignments**	**Peak locations Ex/Em (nm)**	**Number of components**	**Study area (landfill site)**	**Types of waste**	**Types of treatment of MSW**	**Types of landfill**	**References**
**C1**	**Microbial humic-like compounds**	**< 250, 320/395**	**3**	**Japan**	**Incineration residual and incombustible waste**	**Incineration**	**MSW landfill**	**This study**
		250, 320/425	3	Roeselarre (Belgium)			MSW landfill	[14]
		250, 330/417	2	(South Korea)				[21]
		241, 330/410	3	Wuhan (China)				[58]
		240, 320/400	6	Shanghai (China)			Sanitary landfill	[9]
		230, 320/420	5	Shanghai (China)			Lao gang Sanitary Landfill	[28]
		240, 300/420						
		240, 330/412	4	Shanghai (China)	MSW before and after, with or without incineration		MSW sanitary landfill	[8]
		241, 328/405	3	Beijing (China)			Asuwei sanitary landfill	[33]
		255, 285/415	4	Southwest China			A large traditional landfill	[17]
		280, 320/405						
		225, 310/400	3	Incheon, (South Korea)			Sudokwon stabilized landfill site	[20]
		240, 310/410						[27]
		280, 330/390	2	Hunan Province (China)				[47]
**Component**	**Peak assignments**	**Peak locations Ex/Em (nm)**	**Number of components**	**Study area (landfill site)**	**Types of waste**	**Type of treatment of MSW**	**Types of landfill**	**References**
**C2**	**Terrestrial humic-like compounds**	**< 250, 350/450**	**3**	**Japan**	**Incineration residual and incombustible waste**	**Incineration**	**MSW landfill**	**This study**
		250, 310, 370/460	3	Roeselarre (Belgium)			MSW landfill	[14]
		250, 310, 390/458	6	Shanghai (China)			Sanitary landfill	[9]
		255/459	2	(South Korea)				[21]
		250, 360/450	3	Wuhan (China)				[58]
		250, 300, 360/458	4	Shanghai (China)	MSW before and after, with or without incineration		MSW sanitary landfill	[8]
		220, 270, 350/452	6	Shanghai (China)			Sanitary landfill	[9]
		242, 352/452	3	Beijing (China)			Asuwei sanitary landfill	[33]
		250, 310, 360/464	5	Shanghai (China)			Lao gang Sanitary Landfill	[28]
		250, 360/445	4	(Southwest China)			A large traditional landfill	[17]
		275, 348/455	3	Incheon (South Korea)			Sudokwon stabilized landfill site	[20]
		250, 305, 365/458	3					[27]
		250, 360/450	2	Hunan Province (China)				[47]
**Component**	**Peak assignments**	**Peak locations Ex/Em (nm)**	**Number of components**	**Study area (landfill site)**	**Types of waste**	**Type of treatment of MSW**	**Types of landfill**	**References**
**C3**	**Tryptophan-like compounds**	**280/345**	**3**	**Japan**	**Incineration residual and incombustible waste**	**Incineration**	**MSW landfill**	**This study**
		280/322	6	Shanghai (China)			Sanitary landfill	[9]
		270/340	3	Roeselare (Belgium)			MSW landfill	[14]
		230, 280/345	3	Wuhan (China)				[58]
		225, 275/345	4	(Southwest China)			A large traditional landfill	[17]

The OpenFluor database of PARAFAC aquatic fluorescence components (https://openfluor.lablicate.com/; [18]) was used to find matching peaks of EEM-PARAFAC components between the results from this study and those from other studies of a variety of aquatic environments. Matching peaks represented by excitation and emission minimum spectral scores > 0.95 were considered to be similar to the published spectra (S3 Table in S1 File). The fluorescence spectra of C1, C2, and C3 matched 75, 89, and 52 results, respectively, from other studies in the OpenFluor database. The components similar to those in this study originated from various aquatic environments, including rivers [49], streams, groundwater, lakes [50], estuaries, seawater [51], freshwater, aquacultures [52], catchments, wetlands [53], and soil [54]; however, there was a lack of information on FDOM in landfill leachate. Most studies assigned C1 as a marine humic-like or microbial humic-like component, C2 as a terrestrially derived humic-like component, and C3 as a protein or tryptophan-like component. These findings suggest the ubiquitous presence of certain DOM fluorescence spectra across various environments [55] and reinforce the reliability of the three-component PARAFAC model in this study.

Ultimately, the three components were assigned: C1 (Ex/Em < 250, 320/395 nm), microbial humic-like peak M [37, 56]; C2 (Ex/Em < 250, 350/450 nm), a mixture of terrestrial humic-like peaks A and C [37, 57]; and C3 (Ex/Em 280/345 nm), a tryptophan-like peak T [28, 37].

### Spatial and temporal variations in FDOM

**Fig 3:**
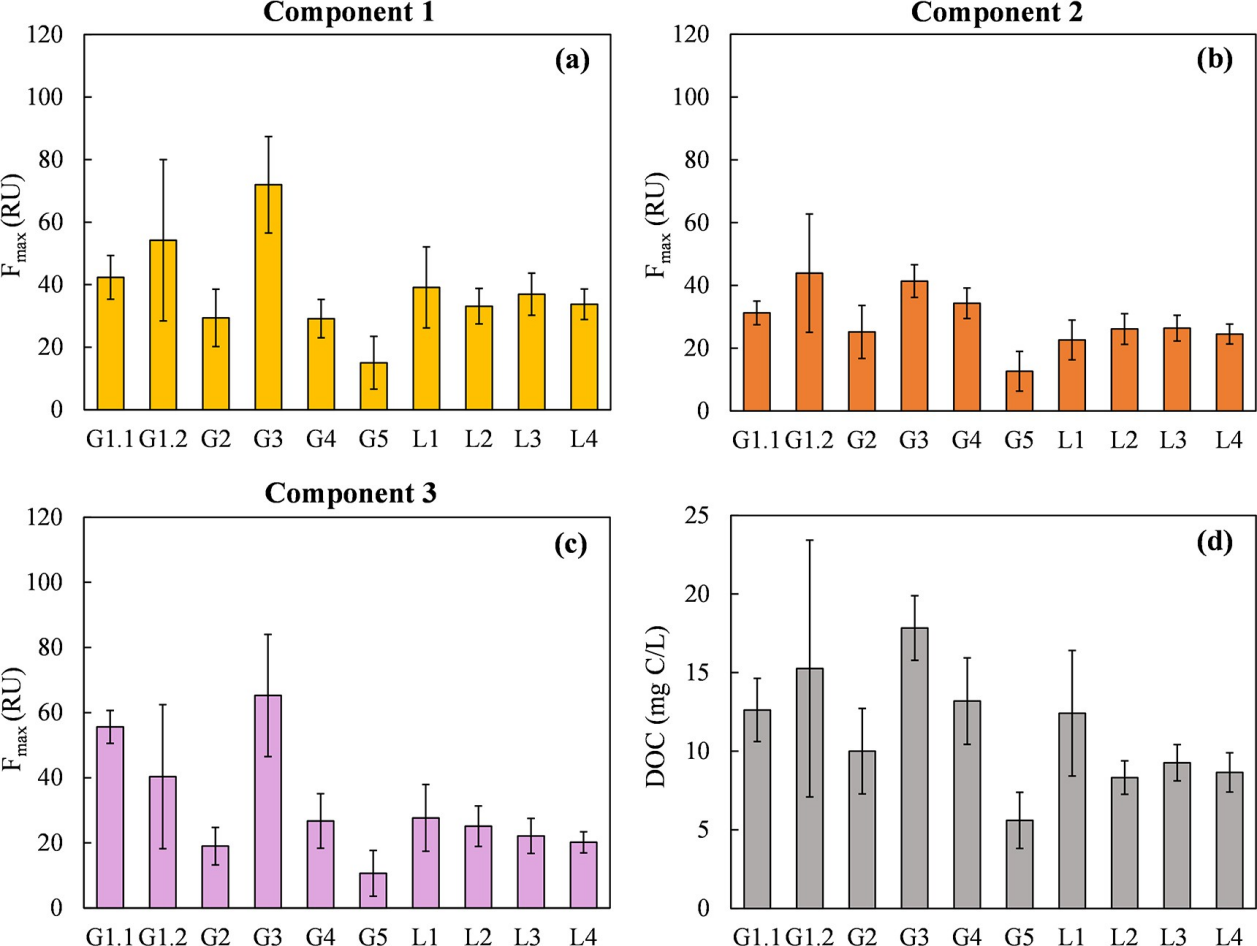
PARAFAC-derived components’ maximum fluorescence intensity variation among samples. (a) Component 1, (b) Component 2, (c) Component 3, and (d) DOC concentration, *(error bars indicate the standard deviation of F*_*max*_ *of (C1*, *C2*, *C3)*, *DOC concentration from June 2019 to June 2020*, *sample size (n = 126))*.

**Fig 4:**
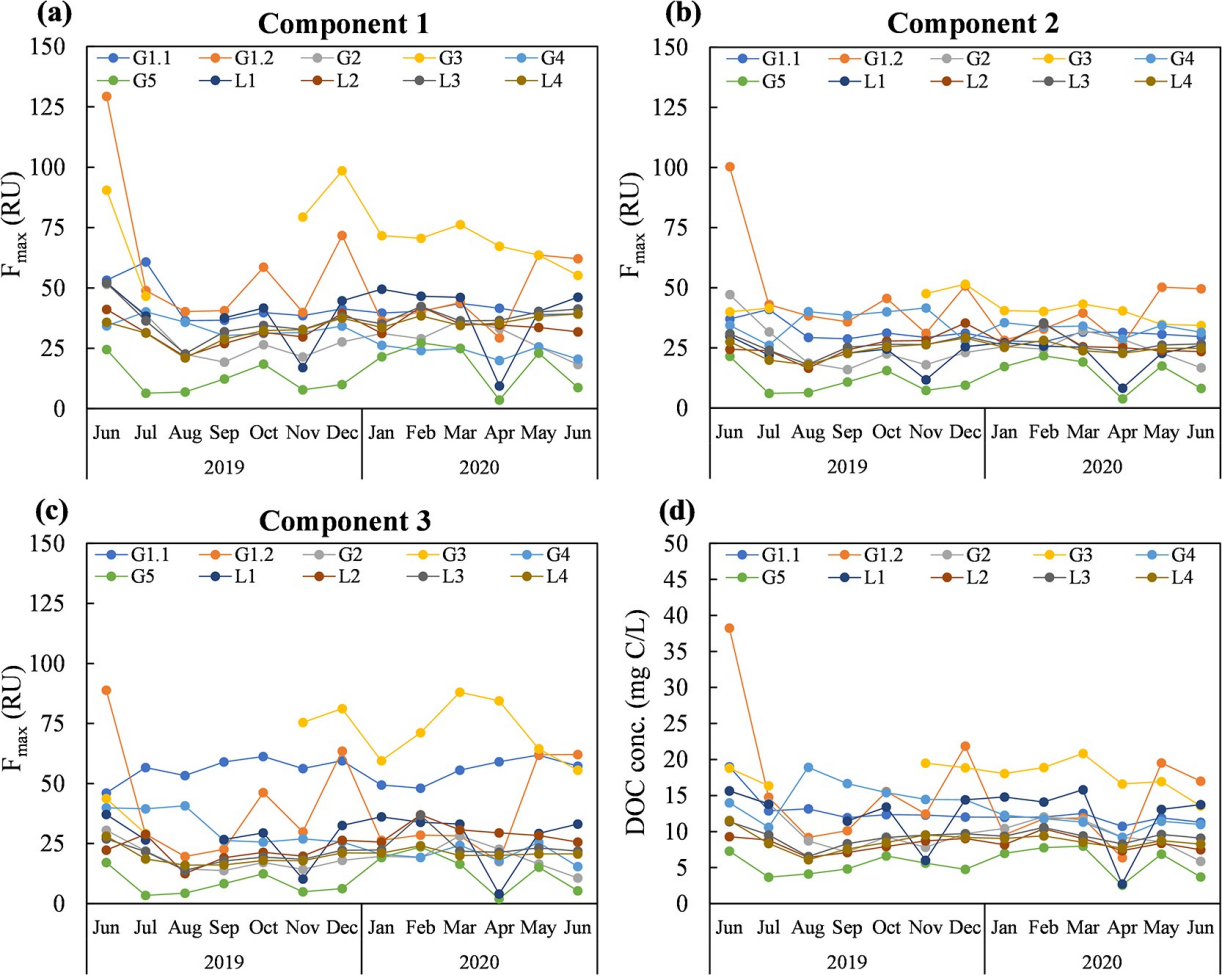
Temporal variation of PARAFAC-derived components’ maximum fluorescence intensity. (a) Component 1, (b) Component 2, (c) Component 3, and (d) DOC concentration.

**Fig 5:**
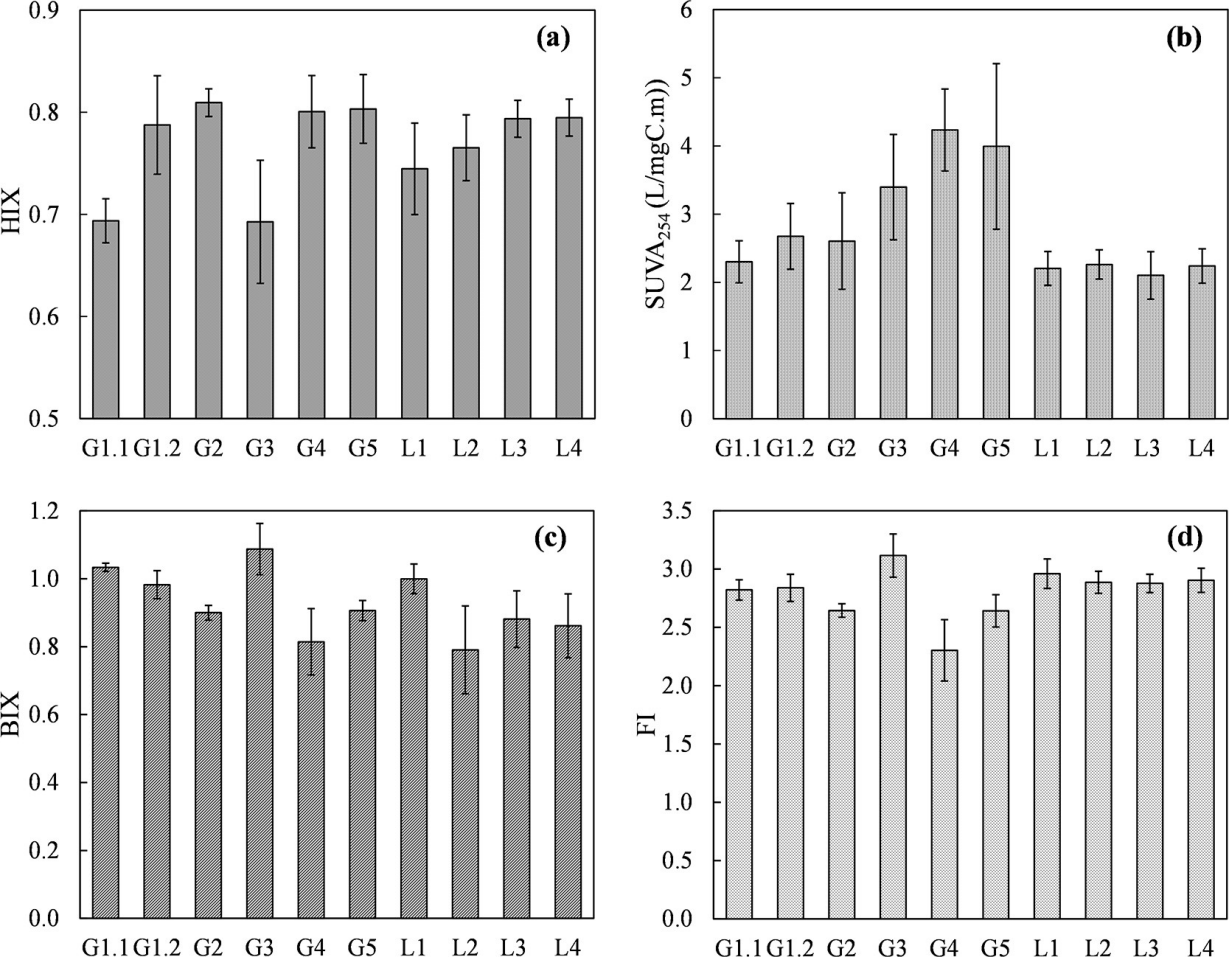
Optical parameters’ spatial variations. (a) Humification index (HIX), (b) Specific UV absorbances (SUVA_254_), (c) Biological index (BIX), (d) Fluorescence index (FI).

Figs 3 and 4 respectively show the spatial and temporal variations in the maximum fluorescence intensities of C1, C2, and C3, as well as the DOC concentrations, of the 10 sampling points from June 2019 to June 2020. The F_max_ of each component is proportional to the concentration in the sample [59]. Fig 5 depicts the variation in optical indices in the same datasets. The DOC concentration of leachate ranged from 2.6 to 38.2 (mg C/L), and the SUVA_254_ ranged from 1.0 to 5.8 (L/mg C·m) (S4 Table in S1 File). Higher DOC concentrations were observed in the samples collected from the old disposal area, including G1.1 (12.6 ± 2.0 mg C/L), G1.2 (15.3 ± 8.2 mg C/L), and G3 (18.4 ± 2.1 mg C/L). Compared with extended disposal area sample G5 (DOC concentration, 5.6 ± 1.8 mg C/L), the F_max_ values of C1 and C3, along with the BIX, indicated a greater influence of microbial and biological activities on FDOM in the old disposal area. Among the sampling sites, G2, G4, and G5 had higher HIX values and lower BIX and FI values (Fig 5). G4 and G5 also had the highest SUVA_254_ values (4.4 ± 0.3 and 4.0 ± 1.2 L/mg C·m, respectively), revealing a higher fraction of aromatic humic substances in the FDOM at these sites (S4 Table in S1 File). Sample G4 had a higher C2 content but lower C1 and C3 contents, indicative of a prominent contribution of terrestrial humic-like components to FDOM. Moreover, samples G2, G4, and G5 had relatively lower C1 and C3 contents. At the leachate treatment facility, the DOM characteristic of sample L1 differed from those of L2, L3, and L4. Site L1, in which leachate was collected from pump wells in the old disposal area and leachate-collecting pipes from sections 4 and 5, had a higher average concentration and variation in the DOC concentration compared with L2, L3, and L4. This was likely the influence of the higher C1 and C3 concentrations in sample G3 from the old disposal area. Overall, the FDOM characteristics differed among sampling sites, in particular between the old and extended disposal areas of the landfill, and between the landfill and leachate treatment facility.

At most sampling sites, the F_max_ values of C1, C2, and C3 followed similar trends over time, with higher values in June 2019, October 2019, December 2019, March 2020, and May 2020 and lower values in August 2019, November 2019, January 2020, and April 2020 (Fig 4). The temporal variation in FDOM was likely partially influenced by variations in precipitation and leachate pump operation, leading to changes in leachate levels.

The leachate treatment method was assessed, with a specific focus on the dissolved organic matter (DOM) characteristics of leachate samples L3 (untreated) and L4 (treated). The F_max_ values of components C1, C2, C3, and DOC concentration, were 36.9 RU, 26.4 RU, 22.1 RU, and 9.26 mg C/L respectively in L3. After treatment (L4), the F_max_ values of C1 and C2, and DOC conc. statistically decreased (Student’s *t*-test, 95% confidence level, *n* = 13 for each parameter) with averaged removal efficiencies over 13 months between L3 and L4 were 8.57%, 7.20%, and 6.4% respectively; however, the F_max_ of C3 was not statistically different after treatment. During the leachate treatment, polyaluminum chloride (PACl) was added as a coagulant to the coagulation-sedimentation process. The coagulation-sedimentation was found to readily remove hydrophobic organic matter with high molecular weight and aromaticity compared to low molecular weight and hydrophilic substances [11, 60]. The presence of weakly acidic functional groups, such as carboxylic groups and phenolic groups, in humic substances may lead to their weak anionic polyelectrolyte-like properties, which, in turn, facilitate the process of flocculation [11, 61]. The analysis of FDOM variations revealed that the F_max_ values of components C1, C2, C3, and DOC concentration in old landfills (G1.1, G1.2, G2, G3, G4), which were operated from 1976–1993, were higher than those observed in extended landfill (G5) operated from 1994–1995 (3.0, 2.8, 3.9, and 2.5 times, respectively). However, compared with the previous study [62] which demonstrated substantial changes in FDOM and ranges of DOC concentration depending on landfill ages, our research revealed relatively low levels of FDOM values and DOC concentrations across 10 different sampling points and 13 sampling months despite the landfill site’s age 25–44 years. Considering the treatment of leachate DOM, these findings suggested it was not necessary to apply complicated or advanced leachate treatment processes. It may be possible to reduce monitoring and treatment activities, especially for leachate treatment in extended landfills, which could help save leachate operational costs.

### Correlation analysis and multilinear regression analysis

**Fig 6:**
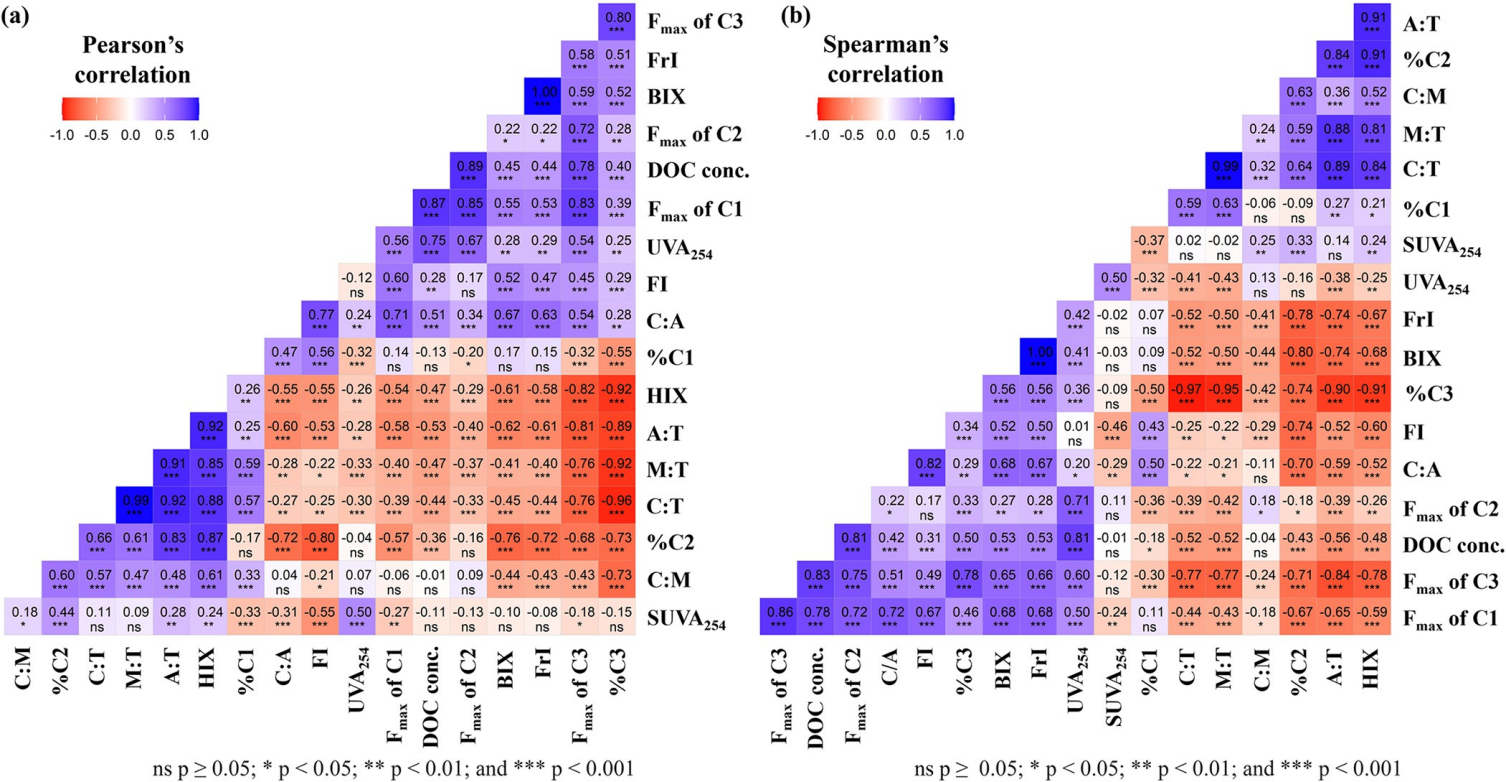
The results of the correlation matrix. (a) Pearson’s r values, (b) Spearman’s rho values.

The results of Pearson’s correlation and Spearman ‘s correlation analysis are presented in Fig 6. The F_max_ values of C1 and C2 were significantly correlated (*r* = 0.85, *ρ* = 0.72, *p <* 0.001, *n* = 126), presumably because both C1 and C2 have similar properties of the humic-like components or potentially originate from similar sources. The F_max_ values of C3 and C1 were also significantly and positively correlated (*r* = 0.83, *ρ* = 0.86, *p* < 0.001). Hudson et al. [63] showed that tryptophan-like components may be present as “free” molecules or may be bound to proteins, peptides, or humic structures. He et al. [64] suggested that the protein-like components in landfill leachate showing a strong correlation with humic-like components were bound mainly to humic-like components with supramolecular assembly structures. Parlanti et al. [65] suggested that co-occurring protein-like peaks and *β* peaks (Ex/Em 310–320/380–420 nm) were derived from biological activity. Thus, the strong correlations between the F_max_ values of C1 and C3 in the landfill leachate in the present study were indicative of an association with microbial or biological activity. This was strengthened by the positive correlations of the BIX with the F_max_ values of C1 (*r* = 0.55, *ρ* = 0.68, *p* < 0.001) and C3 (*r* = 0.59, *ρ* = 0.65, *p* < 0.001). Moreover, the F_max_ of C3 was significantly and negatively correlated with the HIX (*r* = −0.82, *ρ* = 0.78, *p* < 0.001).

In the present study, the SUVA_254_ was not linearly correlated with the F_max_ value of C1 or C2; however, it exhibited a weak positive correlation with %C2 (*r* = 0.44, *ρ* = 0.33, *p* < 0.001). This weak correlation could be due to the complexity of leachate and the variations in leachate characteristics at different sampling points. The presence of iron, colloids, and other constituents in samples may increase the UV absorbance at 254 nm [29, 63]. Broder et al. [66] showed that the DOM origin and dynamics could be tracked using different spectrofluoroscopic indices; however, this method could not display apparent seasonal or hydrologic dynamics in non-fluorescent fractions. The HIX was negatively correlated with F_max_ values of C1 and C2, while it was positively correlated with % C2 (*r* = 0.87, *ρ* = 0.91, *p* < 0.001) and %C1(*r* = 0.26, *ρ* = 0.21, *p* < 0.05). Previous investigations consistently indicated strong positive correlations between the HIX and the relative percentage of terrestrial humic-like components [32, 67, 68], as well as microbially-derived humic-like substances [67, 69], and fulvic acid-like substances [67]. However, Lu et al. [67] reported a negative correlation between HIX and the proportion of microbial humic-like substances (*r* = −0.37, *p* = 0.03, *n* = 35). Gabor et al. [70] noted that fluorescence indices were originally developed for specific sample sets; therefore, they may be suitable only in certain environments, such as marine or soil environments. This could explain the weak correlation between HIX and %C1, which was presumed to originate from microbial activities in our study. Besides, HIX showed strong negative correlation with %C3 (*r* = −0.92, *ρ* = −0.91, *p* < 0.001) and strong positive correlations with A:T, M:T and C:T (*r* = 0.92, 0.85, 0.88, *ρ* = 0.91, 0.81, 0.84, all *p* < 0.001, respectively). He et al. [33] presented correlations among several humification indices and F_max_ values of three PARAFAC components, concluding that several humification indices could not yield a full interpretation of the humic-like components in landfill leachate. However, our findings suggest that HIX and SUVA_254_ should serve as indicators of percentage rather than F_max_ value of terrestrial humic-like components in landfill leachate.

The DOC concentration was significantly, positively correlated with the maximum fluorescence intensities of C1 (*r* = 0.87), C2 (*r* = 0.89), and C3 (*r* = 0.78; all *p* < 0.001). Furthermore, the equation for the relationship between DOC concentration (conc.) and F_max_ values of C2, and C3 can be expressed as follows:

$$DOC\;conc.=-0.01+0.745\times F_{max}\;of\;C2+0.174\times F_{max}\;of\;C3$$ (1)

$$\left(r=0.91,\;R^{2}=0.83,\;p<0.001,\;n=126,\;normalized\;data\right)$$

The variable F_max_ value of C1 was removed from the equation to solve its multicollinearity with other independent variables based on the result of the variance inflation factor (VIF > 5). The result suggested humic-like component has a higher impact on the changes in DOC concentration than the protein-like component. The maximum fluorescence intensity of C2 and C3 could trace DOC concentration, which may contribute to the simplification of DOM measurement. There are few reports on the relationship between DOC and the F_max_ of PARAFAC-derived components in landfill leachate, especially in Japan. However, researchers have investigated the relationship between fluorescence intensity and DOC concentration in freshwater ecosystems [63, 71–73] and marine environments [74]. Different sources of DOM and DOC concentration-related fluorophores result in different gradients (i.e., the fluorescence intensity per gram of carbon) and correlation coefficients of the DOC–fluorescence intensity [71, 72]. The strongest correlation between fluorescence intensity and DOC concentration is typically observed at sites dominated by natural DOM that are less affected by anthropogenic activities [75]. Moreover, a higher fluorescence per gram of carbon was reported in samples dominated by high-molecular-weight aromatic DOM, such as peat catchments and wetlands [71, 72, 75]. In comparison, in the present study, we investigated a site highly influenced by anthropogenic activity, and we measured higher F_max_ values for C1 and C3 than for C2, which had terrestrial humic-like peaks (S10 Fig in S1 File). S11–14 Figs in S1 File shows the relationships of the F_max_ values of C1, C2, and C3 with the DOC concentration, as well as that between the UVA_254_ and DOC concentration, among the samples. These correlations indicated that the temporal variations in the F_max_ values of C1, C2, and C3 were affected by the same factor as the DOC and also highlight the importance of dilution effects. The F_max_ of C2 (terrestrial humic-like component) had a higher positive correlation with the UVA_254_ (*r* = 0.67, *ρ* = 0.71, *p* < 0.001) compared with the other components (S15 Fig in S1 File); however, this relationship was not strong enough to interpret all the changes in humic-like components in the dataset of landfill leachate FDOM.

Several factors may control the characteristics and quantities of FDOM. Stedmon and Cory [48] found that seasonal changes in FDOM characteristics in a catchment were driven by soil microbial activity and rapid drainage of precipitation. Precipitation data were collected at the leachate treatment facility, and seasonal variations in Fmax values of EEM-PARAFAC components and DOC concentration were analyzed; however, no direct statistical effect of precipitation or statistically significant differences among seasons were observed. Therefore, these data were not presented in this research. Since the landfill site was closed and covered by layers of soil, rainfall could penetrate through the soil cover to the waste layers and affect leachate volume. The leachate level was affected by precipitation and leachate pump operation in the landfill. The leachate level was measured from the surface of the leachate to the ground surface (S5 Table in S1 File). The correlation analysis revealed positive correlations of the leachate level with C1 (*r* = 0.57), C2 (*r* = 0.50), and C3 (*r* = 0.41; all *p* < 0.001) across the whole dataset of leachate collected in the landfill (*n* = 72) (S16 Fig in S1 File). These results indicated an influence of a hydrological factor on the variations in the F_max_ values of C1, C2, and C3. For instance, higher leachate levels would be expected to dilute the leachate concentration, although this does not necessarily imply that less precipitation causes higher concentrations of C1, C2, and C3 because pump operation at the leachate treatment facility also affects the leachate level in the landfill. When the leachate level rises significantly during the rainy season, the pumped drainage system is operated to reduce inundation, altering the landfill leachate level.

### Comparison results between EEM-PARAFAC and FRI methods

Furthermore, the findings derived from EEM-PARACFAC concerning the characterization, variations, and proportions of humic-like and protein-like components, as well as their correlations with HIX, BIX, and SUVA_254_, were subjected to a comparative analysis against the results obtained through fluorescence regional integration (FRI). Both approaches yielded comparable findings, allowing for a rough estimation of FDOM variations. However, EEM-PARAFAC exhibited advantages in resolving FDOM into distinct fluorescent components and mitigating peak overlap. Further details on these findings are provided in the S6 Text, S6 Table, S17 Fig of S1 File.

## Conclusions

We evaluated the DOM characteristics in the leachate of an old landfill in Japan from June 2019 to June 2020 using EEM-PARAFAC and correlation analysis. The DOC concentration ranged from 2.6 to 38.2 mg C/L and the SUVA_254_ from 1.0 to 5.8 L/mg C·m. EEM-PARAFAC identified three fluorescent components: C1, microbial humic-like compounds; C2, terrestrial humic-like compounds; and C3, tryptophan-like compounds. The FDOM of landfill leachate varied spatially among the old disposal area, extended disposal area, and leachate treatment facility. Temporal trends showed particularly pronounced changes in August 2019, November 2019, and April 2020. Leachate DOM was assessed before and after treatment, indicating lower levels of DOM compared to leachate from other landfills. The findings may contribute to selecting suitable leachate treatment methods and landfill site management strategies, especially when considering the increasing popularity of incineration for waste management. The characterization of DOM in leachate from landfills containing incineration residues is highly relevant in this context. Correlation analysis supported the possible origin of EEM-PARAFAC components related to microbial and biological activity or terrestrial matter. F_max_ values of C1-C3 showed strong correlations with DOC, a novel finding in landfill leachate that may simplify DOM measurement. The strong positive correlation between HIX and %C2 suggests that HIX could serve as an indicator of the percentage of terrestrial humic-like components in landfill leachate. The findings help characterize FDOM and clarify its relationship with optical indices and DOC concentration in the leachate of a landfill containing incineration residues and incombustible waste, which is different from previous studies. The study may provide a useful reference when comparing DOM quality and FDOM properties in leachate in different contexts.

Our findings revealed that fluorescence spectroscopy is a useful, sensitive, and quick analytical technique for identifying and monitoring variations in different FDOM components in this specific type of leachate. Future work on fractional quantification and characterizations of leachate’s FDOM based on molecular weight is necessary to enhance our understanding of the environmental behavior of leachate, given its heterogeneity. Hereafter, based on the spatiotemporal distribution of FDOM screened and demonstrated in this research, we plan to evaluate DOM at the molecular level, chemical structural analysis (e.g., Orbitrap-LC-MS/MS) and the effects of DOM on the behavior of toxic chemicals, based on the conclusions drawn from this study.

## Supporting information

S1 File (DOCX)

## References

[1] Cossu R, Stegmann R. Solid waste landfilling: Concepts, processes, technology. Elsevier; 2019.

[2] Xiong Y, Takaoka M, Sano A, Kusakabe T, Yang J, Shiota K, et al. Distribution and characteristics of heavy metals in a first-generation monofill site for incinerator residue. J Hazard Mater. 2019; 373: 763–772. doi: 10.1016/j.jhazmat.2019.04.019 30965241

[3] Istrate IR, Galvez-Martos JL, Dufour J. The impact of incineration phase-out on municipal solid waste landfilling and life cycle environmental performance: Case study of Madrid, Spain. Sci Total Environ. 2021; 755: 142537. doi: 10.1016/j.scitotenv.2020.142537 33035976

[4] Seo DJ, Kim YJ, Ham SY, Lee DH. Characterization of dissolved organic matter in leachate discharged from final disposal sites which contained municipal solid waste incineration residues. J Hazard Mater. 2007; 148(3): 679–692. doi: 10.1016/j.jhazmat.2007.03.027 17452075

[5] Jiang Y, Li R, Yang Y, Yu M, Xi B, Li M, et al. Migration and evolution of dissolved organic matter in landfill leachate-contaminated groundwater plume. Resour Conserv Recycl. 2019; 151: 104463. doi: 10.1016/j.resconrec.2019.104463

[6] Ma Y, Liu ZH, Xi BD, Li WT, Xu YQ, Zhao HZ, et al. Molecular structure and evolution characteristics of dissolved organic matter in groundwater near landfill: implications of the identification of leachate leakage. Sci Total Environ. 2021; 787: 147649. doi: 10.1016/j.scitotenv.2021.147649 34000547

[7] Xiong Y, Takaoka M, Kusakabe T, Shiota K, Oshita K, Fujimori T. Mass balance of heavy metals in a non-operational incinerator residue landfill site in Japan. J Mater Cycles Waste Manag. 2020; 22: 354–364. doi: 10.1007/s10163-020-00976-w

[8] Wu J, Zhang H, He PJ, Shao LM. Insight into the heavy metal binding potential of dissolved organic matter in MSW leachate using EEM quenching combined with PARAFAC analysis. Water Res. 2011; 45(4): 1711–1719. doi: 10.1016/j.watres.2010.11.022 21163510

[9] Wu J, Zhang H, Shao LM, He PJ. Fluorescent characteristics and metal binding properties of individual molecular weight fractions in municipal solid waste leachate. Environ Pollut. 2012; 162: 63–71. doi: 10.1016/j.envpol.2011.10.017 22243848

[10] He PJ, Xue JF, Shao LM, Li GJ, Lee DJ. Dissolved organic matter (DOM) in recycled leachate of bioreactor landfill. Water Res. 2006; 40(7), 1465–1473. doi: 10.1016/j.watres.2006.01.048 16546235

[11] Japanese International Cooperation Agency (JICA). Japan’s Experiences on Waste Management. 2022. Available from: https://openjicareport.jica.go.jp/pdf/12380333_01.pdf

[12] Murphy KR, Stedmo CA, Graeber D, Bro R. Fluorescence spectroscopy and multi–way techniques. PARAFAC. Anal Methods. 2013; 5(23): 6557–6566. doi: 10.1039/c3ay41160e

[13] Yang L, Hur J, Zhuang W. Occurrence and behaviors of fluorescence EEM-PARAFAC components in drinking water and wastewater treatment systems and their applications: a review. Environ Sci Pollut Res. 2015; 22: 6500–6510. doi: 10.1007/s11356-015-4214-3 25854204

[14] Oloibiri V, De Coninck S, Chys M, Demeestere K, Van Hulle SW. Characterisation of landfill leachate by EEM-PARAFAC-SOM during physical-chemical treatment by coagulation-flocculation, activated carbon adsorption and ion exchange. Chemosphere. 2017; 186: 873–883. doi: 10.1016/j.chemosphere.2017.08.035 28826135

[15] Xu X, Kang J, Shen J, Zhao S, Wang B, Zhang X, et al. EEM–PARAFAC characterization of dissolved organic matter and its relationship with disinfection by-products formation potential in drinking water sources of northeastern China. Sci Total Environ. 2021; 774: 145297. doi: 10.1016/j.scitotenv.2021.145297 33611000

[16] Henderson RK, Baker A, Murphy KR, Hambly A, Stuetz RM, Khan SJ (2009). Fluorescence as a potential monitoring tool for recycled water systems: a review. Water Res. 2009; 43(4): 863–881. doi: 10.1016/j.watres.2008.11.027 19081598

[17] Chen W, Zhang A, Jiang G, Li Q. Transformation and degradation mechanism of landfill leachates in a combined process of SAARB and ozonation. Waste Manag. 2019; 85: 283–294. doi: 10.1016/j.wasman.2018.12.038 30803582

[18] Murphy KR, Stedmon CA, Wenig P, Stedmon R. OpenFluor–A spectral database of auto-fluorescence by organic compounds in the environment. Anal Methods. 2014; 6(3): 658–661. doi: 10.1039/c3ay41935e

[19] Andersen CM, Bro R. Practical aspects of PARAFAC modeling of fluorescence excitation‐emission data. J Chemom. 2003; 17(4): 200–215. doi: 10.1002/cem.790

[20] Aftab B, Cho J, Shin HS, Hur J. Using EEM–PARAFAC to probe NF membrane fouling potential of stabilized landfill leachate pretreated by various options. Waste Manag. 2020; 102: 260–269. doi: 10.1016/j.wasman.2019.10.035 31693970

[21] Lee S, Hur J. Heterogeneous adsorption behavior of landfill leachate on granular activated carbon revealed by fluorescence excitation emission matrix (EEM)–parallel factor analysis (PARAFAC). Chemosphere. 2016; 149: 41–48. doi: 10.1016/j.chemosphere.2016.01.081 26849193

[22] Han Z, Xiao M, Yue F, Yi Y, Mostofa KMG. Seasonal variations of dissolved organic matter by fluorescent analysis in a typical river catchment in Northern China. Water. 2021; 13(4): 494. doi: 10.3390/w13040494

[23] Wen Y, Xiao M, Chen Z, Zhang W, Yue F. Seasonal variations of dissolved organic matter in urban rivers of Northern China. Land. 2023; 12(2): 273. doi: 10.3390/land12020273

[24] Liu M, Tian H, Chen T, Sun J, Sun R, Kong Q, et al. Spatiotemporal evolution of dissolved organic matter (DOM) and its response to environmental factors and human activities. Plos One. 2023; 18(10): e0292705. doi: 10.1371/journal.pone.0292705 37819935 PMC10566700

[25] Yang X, Yuan J, Yue FJ, Li SL, Wang B, Mohinuzzaman M, et al. New insights into mechanisms of sunlight-and dark-mediated high-temperature accelerated diurnal production-degradation of fluorescent DOM in lake waters. Sci Total Environ. 2021; 760: 143377. doi: 10.1016/j.scitotenv.2020.143377 33198994

[26] Wang Y, Ren D, Li Y, Hao Z, Liu J. Spatiotemporal dynamics of dissolved organic matter and disinfection by-products formation potential of Shengzhong Lake in southwest China. Environ Sci and Pollut Res. 2024; 1–10. doi: 10.1007/s11356-024-32548-y 38393559

[27] Aftab B, Ok YS, Cho J, Hur J. Targeted removal of organic foulants in landfill leachate in forward osmosis system integrated with biochar/activated carbon treatment. Water Res. 2019; 160: 217–227. doi: 10.1016/j.watres.2019.05.076 31152947

[28] Lu F, Chang CH, Lee DJ, He PJ, Shao LM, Su A. Dissolved organic matter with multi–peak fluorophores in landfill leachate. Chemosphere. 2009; 74(4): 575–582. doi: 10.1016/j.chemosphere.2008.09.060 18986674

[29] Weishaar JL, Aiken GR, Bergamaschi BA, Fram MS, Fujii R, Mopper K. Evaluation of specific ultraviolet absorbance as an indicator of the chemical composition and reactivity of dissolved organic carbon. Environ Sci Technol. 2003; 37(20): 4702–4708. doi: 10.1021/es030360x 14594381

[30] Ohno T. Fluorescence inner-filtering correction for determining the humification index of dissolved organic matter. Environ Sci Technol. 2002; 36(4): 742–746. doi: 10.1021/es0155276 11878392

[31] Zhao R, Li Z, Aye AA, Zheng H, Jin W, Zhang J, et al. Characteristics of chromophoric dissolved organic matter in the Northern Andaman Sea. Front Mar Sci. 2023; 9: 1043194. doi: 10.3389/fmars.2022.1043194

[32] Bai L, Liu X, Wu Y, Cheng H, Wang C, Jiang H, et al. Distinct seasonal variations of dissolved organic matter across two large freshwater lakes in China: Lability profiles and predictive modeling. J Environ Manag. 2023; 339: 117880. doi: 10.1016/j.jenvman.2023.117880 37080098

[33] He XS, Xi BD, Li X, Pan HW, An D, Bai SG, et al. Fluorescence excitation–emission matrix spectra coupled with parallel factor and regional integration analysis to characterize organic matter humification. Chemosphere. 2013; 93(9): 2208–2215. doi: 10.1016/j.chemosphere.2013.04.039 23706894

[34] Teng C, Zhou K, Peng C, Chen W. Characterization and treatment of landfill leachate: A review. Water Res. 2021; 203: 117525. doi: 10.1016/j.watres.2021.117525 34384952

[35] 一般財団法人日本規格協会. JIS K 0102: 2016, 工場排水試験方法. JISハンドブック. 2019. Japanese.

[36] Stedmon CA. Thomas DN, Granskog M, Kaartokallio H, Papadimitriou S, Kuosa H. Characteristics of Dissolved Organic Matter in Baltic Coastal Sea Ice: Allochthonous or Autochthonous Origins? Environ Sci Technol. 2007; 41(21): 7273–7279. doi: 10.1021/es071210f 18044499

[37] Chen W, Westerhoff P, Leenheer JA, Booksh K. Fluorescence excitation− emission matrix regional integration to quantify spectra for dissolved organic matter. Environ Sci Technol. 2003; 37(24): 5701–5710. doi: 10.1021/es034354c 14717183

[38] Bro R, Kiers HA. A new efficient method for determining the number of components in PARAFAC models. J Chemom. 2003; 17(5): 274–286. doi: 10.1002/cem.801

[39] Riu J, Bro R. Jack-knife technique for outlier detection and estimation of standard errors in PARAFAC models. Chemom Intell Lab. 2003; 65(1): 35–49. doi: 10.1016/S0169-7439(02)00090-4

[40] Hansen AM, Kraus TE, Pellerin BA, Fleck JA, Downing BD, Bergamaschi BA. Optical properties of dissolved organic matter (DOM): Effects of biological and photolytic degradation. Limnol Oceanogr. 2016; 61(3): 1015–1032. doi: 10.1002/lno.10270

[41] McKnight DM, Boyer EW, Westerhoff PK, Doran PT, Kulbe T, Andersen DT. Spectrofluorometric characterization of dissolved organic matter for indication of precursor organic material and aromaticity. Limnol Oceanogr. 2001; 46(1): 38–48. doi: 10.4319/lo.2001.46.1.0038

[42] Cory RM, McNeill K, Cotner JP, Amado A, Purcell JM, Marshall A. Singlet oxygen in the coupled photochemical and biochemical oxidation of dissolved organic matter. Environ Sci Technol. 2010; 44(10): 3683–3689. doi: 10.1021/es902989y 20408544

[43] Huguet A, Vacher L, Relexans S, Saubusse S, Froidefond JM, Parlanti E. Properties of fluorescent dissolved organic matter in the Gironde Estuary. Org Geochem. 2009; 40(6): 706–719. doi: 10.1016/j.orggeochem.2009.03.002

[44] Hur J, Cho J. Prediction of BOD, COD, and total nitrogen concentrations in a typical urban river using a fluorescence excitation-emission matrix with PARAFAC and UV absorption indices. Sensors. 2012; 12(1): 972–986. doi: 10.3390/s120100972 22368505 PMC3279249

[45] He XS, Fan QD. Investigating the effect of landfill leachates on the characteristics of dissolved organic matter in groundwater using excitation–emission matrix fluorescence spectra coupled with fluorescence regional integration and self-organizing map. Environ Sci Pollut Res. 2016; 23: 21229–21237. doi: 10.1007/s11356-016-7308-7 27491518

[46] Yang X, Meng L, Meng F. Combination of self-organizing map and parallel factor analysis to characterize the evolution of fluorescent dissolved organic matter in a full-scale landfill leachate treatment plant. Sci Total Environ. 2019; 654: 1187–1195. doi: 10.1016/j.scitotenv.2018.11.135 30841393

[47] Zhang Z, Teng C, Zhou K, Peng C, Chen W. Degradation characteristics of dissolved organic matter in nanofiltration concentrated landfill leachate during electrocatalytic oxidation. Chemosphere. 2020; 255: 127055. doi: 10.1016/j.chemosphere.2020.127055 32679637

[48] Jung C, Deng Y, Zhao R, Torrens K. Chemical oxidation for mitigation of UV-quenching substances (UVQS) from municipal landfill leachate: fenton process versus ozonation. Water Res. 2017; 108: 260e270. doi: 10.1016/j.watres.2016.11.005 27836172

[49] Logozzo LA, Hosen JD, McArthur J, Raymond PA. Distinct drivers of two size fractions of operationally dissolved iron in a temperate river. Limnology and Oceanography. 2023; 68(6): 1185–1200. doi: 10.1002/lno.12338

[50] Kothawala DN, Von Wachenfeldt E, Koehler B, Tranvik LJ. Selective loss and preservation of lake water dissolved organic matter fluorescence during long-term dark incubations. Sci Total Environ. 2012; 433, 238–246. doi: 10.1016/j.scitotenv.2012.06.029 22796414

[51] Yamashita Y, Cory RM, Nishioka J, Kuma K, Tanoue E, Jaffé R. Fluorescence characteristics of dissolved organic matter in the deep waters of the Okhotsk Sea and the northwestern North Pacific Ocean. Deep Sea Res Part II: Top Stud Oceanogr. 2010; 57(16): 1478–1485. doi: 10.1016/j.dsr2.2010.02.016

[52] Hambly AC, Arvin E, Pedersen LF, Pedersen PB, Seredyńska-Sobecka B, Stedmon CA. Characterising organic matter in recirculating aquaculture systems with fluorescence EEM spectroscopy. Water Res. 2015; 83: 112–120. doi: 10.1016/j.watres.2015.06.037 26141427

[53] Yan C, Wang W, Nie M, Ding M, Wang P, Zhang H, et al. Characterization of copper binding to biochar-derived dissolved organic matter: effects of pyrolysis temperature and natural wetland plants. J Hazard Mater. 2023; 442: 130076. doi: 10.1016/j.jhazmat.2022.130076 36193612

[54] Sharma P, Laor Y, Raviv M, Medina S, Saadi I, Krasnovsky A, et al. Compositional characteristics of organic matter and its water-extractable components across a profile of organically managed soil. Geoderma. 2017; 286: 73–82. doi: 10.1016/j.geoderma.2016.10.014

[55] Wünsch UJ, Murphy K. A simple method to isolate fluorescence spectra from small dissolved organic matter datasets. Water Res. 2021; 190: 116730. doi: 10.1016/j.watres.2020.116730 33348069

[56] Coble PG. Characterization of marine and terrestrial DOM in seawater using excitation–emission matrix spectroscopy. Mar Chem. 1996; 51(4): 325–346. doi: 10.1016/0304-4203(95)00062-3

[57] Stedmon CA, Markager S. Resolving the variability in dissolved organic matter fluorescence in a temperate estuary and its catchment using PARAFAC analysis. Limnology and oceanography. 2005; 50(2): 686–697. doi: 10.4319/lo.2005.50.2.0686

[58] Ye Z, Zhang H, Zhang X, Zhou D. Treatment of landfill leachate using electrochemically assisted UV/chlorine process: effect of operating conditions, molecular weight distribution and fluorescence EEM–PARAFAC analysis. J Chem Eng. 2016; 286: 508–516. doi: 10.1016/j.cej.2015.10.017

[59] Stedmon CA, Bro R. Characterizing dissolved organic matter fluorescence with parallel factor analysis: a tutorial. Limnol Oceanogr: Methods. 2008; 6(11), 572–579. doi: 10.4319/lom.2008.6.572

[60] Phatthalung WN, Suttinun O, Phungsai P, Kasuga I, Kurisu F, Furumai H, et al. Non-target screening of dissolved organic matter in raw water, coagulated water, and chlorinated water by Orbitrap mass spectrometry. Chemosphere. 021; 264: 128437. doi: 10.1016/j.chemosphere.2020.128437 33045510

[61] O’melia CR, Becker WC, Au K K. Removal of humic substances by coagulation. Water Sci Technol. 1999;40(9): 47–54. doi: 10.2166/wst.1999.0440

[62] Shouliang HUO, Beidou XI, Haichan YU, Liansheng HE, Shilei FAN, Hongliang LIU. Characteristics of dissolved organic matter (DOM) in leachate with different landfill ages. J Environ Sci. 2008; 20(4): 492–498. doi: 10.1016/s1001-0742(08)62085-9 18575137

[63] Hudson N, Baker A, Reynolds D. Fluorescence analysis of dissolved organic matter in natural, waste and polluted waters—a review. River Res Appl. 2007; 23(6): 631–649. doi: 10.1002/rra.1005

[64] He XS, Xi BD., Gao RT, Zhang H, Dang QL, Li D, Huang CH. Insight into the composition and degradation potential of dissolved organic matter with different hydrophobicity in landfill leachates. Chemosphere. 2016; 144, 75–80. doi: 10.1016/j.chemosphere.2015.08.071 26347928

[65] Parlanti E, Wörz K, Geoffroy L, Lamotte M. Dissolved organic matter fluorescence spectroscopy as a tool to estimate biological activity in a coastal zone submitted to anthropogenic inputs. Organic geochemistry. 2000; 31(12): 1765–1781. doi: 10.1016/S0146-6380(00)00124-8

[66] Broder T, Knorr KH, Biester H. Changes in dissolved organic matter quality in a peatland and forest headwater stream as a function of seasonality and hydrologic conditions. Hydrol Earth Syst Sci. 2017; 21(4): 2035–2051. doi: 10.5194/hess-21-2035-2017

[67] Lu Y, Shang P, Chen S, Du Y, Bonizzoni M, Ward AK. Discharge and temperature controls of dissolved organic matter (DOM) in a forested coastal plain stream. Water. 2021; 13(20): 2919. doi: 10.3390/w13202919

[68] Badalge NDK, Kim J, Lee S, Lee BJ, Hur J. Land use effects on spatiotemporal variations of dissolved organic matter fluorescence and water quality parameters in watersheds, and their interrelationships. J Hydrol. 2024; 631: 130840. doi: 10.1016/j.jhydrol.2024.130840

[69] Li M, Xie W, Li P, Yin K, Zhang C. Establishing a terrestrial proxy based on fluorescent dissolved organic matter from sediment pore waters in the East China Sea. Water Res. 2020; 182: 116005. doi: 10.1016/j.watres.2020.116005 32645457

[70] Gabor RS, Baker A, McKnight DM, Miller MP. Fluorescence indices and their interpretation. Aquatic organic matter fluorescence. Cambridge University Press; 2014. pp 303–338. doi: 10.1017/CBO9781139045452.015

[71] Baker A, Bolton L, Newson M, Spencer RG. Spectrophotometric properties of surface water dissolved organic matter in an afforested upland peat catchment. Hydrol Process. 2008; 22(13): 2325–2336. doi: 10.1002/hyp.6827

[72] Cumberland SA, Baker A. The freshwater dissolved organic matter fluorescence–total organic carbon relationship. Hydrol Process. 2007; 21(16), 2093–2099. doi: 10.1002/hyp.6371

[73] Baker A. Fluorescence excitation− emission matrix characterization of river waters impacted by a tissue mill effluent. Environ Sci Technol. 2002; 36(7): 1377–1382. doi: 10.1021/es0101328 11999038

[74] Kowalczuk P, Cooper WJ, Durako MJ, Kahn AE, Gonsior M, Young H. Characterization of dissolved organic matter fluorescence in the South Atlantic Bight with use of PARAFAC model: Relationships between fluorescence and its components, absorption coefficients and organic carbon concentrations. Mar Chem. 2010; 118(1–2), 22–36. doi: 10.1016/j.marchem.2009.01.015

[75] Coble PG, Lead J, Baker A, Reynolds DM, Spencer RG. ed. Aquatic organic matter fluorescence. Cambridge University Press; 2014. doi: 10.1017/CBO9781139045452

