# Supplementary material for: Characterization and spatiotemporal variations of fluorescent dissolved organic matter in leachate from old landfill-derived incineration residues and incombustible waste
Source: PLoS One. 2024 Jun 26;19(6):e0304188. doi: 10.1371/journal.pone.0304188 (PMC11207158; doi:10.1371/journal.pone.0304188)
Supplement: S1 File — (DOCX) [file pone.0304188.s001.docx]

**Supporting information for**

**Characterization and spatiotemporal variations of fluorescent dissolved organic matter in leachate from old landfill-derived incineration residues and incombustible waste**

Thi Ngoc Nguyen ^1^, Taketoshi Kusakabe ^1,#a^, Masaki Takaoka ^1*^

^1^ Department of Environmental Engineering, Graduate School of Engineering, Kyoto University,

C-cluster, Katsura campus, Nishikyo-ku, Kyoto, 615-8540, Japan

^#a^ Current Address: Department of Environmental Engineering, Faculty of Engineering, Osaka Institute of Technology, 5-16-1 Ohmiya, Asahi-ku, Osaka, 535-8585, Japan

*Corresponding author:

E-mail: takaoka.masaki.4w@kyoto-u.ac.jp (Masaki Takaoka)

TEL: +81-75-383-3335

FAX: +81-75-383-3338

Submitted to ***PLOS ONE***

**Summary:**

Number of pages: 36

Number of texts: 6

Number of tables: 6

Number of figures: 17

Number of equations: 6

# **S1 Text. Inner filter effect correction (IFE)**

The primary IFE is incident light absorbed before luminescence occurs, and secondary IFEs occur when emitted light is re-absorbed in a sample, and the fluorescence intensity is not proportional to the concentration of fluorophores [1]. To correct for IFEs, the samples were diluted up to 40-fold (depending on the sample) using ultrapure water acidified to pH 2.0 ± 0.1 with HCl, and the following UV absorbance-based formulation was applied for samples collected from August 2019 to June 2020 [2, 3]. The IFEs of the samples collected in June and July 2019 were corrected by applying only the dilution method at the absorbance, at which the difference in fluorescence intensity of the diluted samples, before and after applying the UV absorbance-based formulation was less than 5%.

$F_{corr}=F_{obs}{10}^{\frac{{Abs}_{ex} + {Abs}_{em}}{2}}$ (1)

Here, *F_corr_* and *F_obs_* refer to the corrected and observed fluorescence intensities, respectively; *Abs_ex_* and *Abs_em_* denote the absorbance at the corresponding excitation wavelength and emission wavelength, respectively. The absorbance-based equation corrects for IFEs in DOM samples when the total absorbance (*Abs_ex_* + *Abs_em_*) is below 1.5 in a 1-cm cuvette [2]

# **S2 Text. PARAFAC**

A PARAFAC model of a three-way array is given by three loading matrices, A, B, and C, with elements *a_if_, b_jf_,* and *c_kf_.* This trilinear model minimizes the sum of squares of the residuals, *ε_ijk_*, in the model. This chemometric decomposition method uses alternating least squares algorithms to estimate the underlying components of a multiway dataset [4]:

$x_{ijk}=\sum_{f=1}^{F} a_{if}b_{jf}c_{kf}+\varepsilon_{ijk}$*,* *i =1,.,I*; *j = 1,.,J*; *k =1,.,K*　(2)

Here, *x_ijk_* represents an element of the three-way data array with dimensions *I*, *J*, and *K*. In the analysis of EEMs, *x_ijk_* is the fluorescence intensity of the *i*th sample measured at emission wavelength *j* and excitation wavelength *k*. *F* is the number of factors or components; *I* is the number of samples; *J* is the emission wavelength; and *K* is the excitation wavelength. Finally, *ε_ijk_* is the unexplained signal (residuals containing noise and other unmodeled variations). The model outputs are the parameters *a*, *b*, and *c*. Ideally, these represent the score values, emission loadings, and excitation loadings of the underlying fluorophores, respectively [5].

# **S3 Text. Model validations**

Three model validation approaches (residuals and loadings analysis, core consistency, and split-half analysis) were evaluated. The residual sum of squares decreased as the number of model components increased from 2 to 7, indicating that more components yielded better model fits. The two-component model had the highest sum of squares errors and was unlikely to be a suitable candidate (Fig. S1). Bro and Kiers [6] recommended the core consistency diagnostic to determine the appropriate number of components in PARAFAC based on evaluating the “appropriateness” of the structural model. When a sequence of gradually augmented component models is run, the core consistency tends to level off slowly from the high value (near 100%) and then sharply declines when the optimal number of components is exceeded. A core consistency close to zero, or even negative, implies that the data cannot be described primarily by the trilinear model, or that too many components have been included [6, 7]. The number of components that should be included corresponds to the largest model with a high core consistency value [7]. In this research, the core consistency and number of component plots in all cases depicted high core consistency values for the two- and three-component models (> 84%) and then sharply decreased for the four-, five-, six-, and seven-component models (< 3%) (Fig. S2). This trend indicated that the models containing four to seven components were not stable; hence, according to the core consistency, the three-component model should be selected. In the split-half analysis, the dataset was split into four groups, and four quarter splits were combined into six dataset halves to produce three validation tests [7]. Comparing the components in the split models in each test matched a Tucker congruence coefficient > 0.95 [8]. The Tucker congruence coefficient has been described as an index of similarity [9] and was used to assess emission and excitation spectral congruences among components in this research. The results showed that all comparisons in the split-half analysis were validated for the two-, three-, four-, and six-component models but not for the five- and seven-component models (Fig. S3a–f). Thus, all three validation approaches (residuals and loadings analysis, core consistency, and split-half analysis) indicated that the three-component model was optimal.

Finally, the results were obtained as the maximum fluorescence intensity, *x_ijk_*, and excitation and emission loadings for each sample.

# **S4 Text. Split-half analysis results**

Data were split into 4 groups named A, B, C, and D, and then four-quarter splits were combined into six data halves 'AB', 'CD', 'AC', 'BD', 'AD', and 'BC'. After that, six halves were compared in each pair producing 3 validation tests (1 2; 3 4; 5 6). The excitation and emission spectra from PARAFAC models of splits were compared based on the Tucker congruence coefficient (TCC) to determine the similarity of two pairs of excitation and emission spectrum [7, 9]. Furthermore, each split in the validation will be compared with the overall model. If splits contain independent datasets and components are congruent among various splits, the model is validated [7].

ExCC: Tucker congruency coefficients for Ex spectra for the nth comparison is in ExCC{n}

EmCC: Tucker congruency coefficients for Em spectra for the nth comparison is in EmCC{n}

R: a cell structure with the matched components tabulated in R{1} (1st comparison), R{2} (2nd comparison), etc.

Xpre: normalized dataset

LSmodel3: A structure containing only the 3-component model that had the least squares solution with random initialization and convergence criterion 1e-08

LSmodel3r: Reverse normalization of LSmodel3 to obtain the unscaled scores.

>> S1=splitds(Xpre,[],4,'alternating',{[1 2],[3 4],[1 3],[2 4],[1 4],[2 3]});

>> A1=psplitanalysis(S1,2:7,'nonnegativity');

**Split-half analysis result of two-component model**

>> val2=splitvalidation(A1,2,[1 2;3 4;5 6],{'AB','CD','AC','BD','AD','BC'},LSmodel2r);

*Output:*

Overall Result= Validated for all comparisons

**Split-half analysis result of three-component model**

>> val3=splitvalidation(A1,3,[1 2;3 4;5 6],{'AB','CD','AC','BD','AD','BC'},LSmodel3r);

*Output:*

Overall Result= Validated for all comparisons

**Split-half analysis result of four-component model**

>> val4=splitvalidation(A1,4,[1 2;3 4;5 6],{'AB','CD','AC','BD','AD','BC'},LSmodel4r);

*Output:*

Overall Result= Validated for all comparisons

**Split-half analysis result of five-component model**

>> val5=splitvalidation(A1,5,[1 2;3 4;5 6],{'AB','CD','AC','BD','AD','BC'},LSmodel5r);

*Output:*

Overall Result = Not Validated

In the following tables, Match = 1, No Match = 0

Rows and columns are interpreted as follows:

Rows: depict the 1st model in each comparison

Columns: depict the 2nd model in each comparison

Model 5:AB vs CD-Validated

Model 5:AC vs BD-Validated

Model 5:AD vs BC-Not Validated

0 0 0 0 0

0 0 0 0 0

0 0 0 1 0

0 0 0 0 1

0 0 0 0 0

**Split-half analysis result of six-component model**

>> val6=splitvalidation(A1,6,[1 2;3 4;5 6],{'AB','CD','AC','BD','AD','BC'},LSmodel6r);

*Output:*

Overall Result= Validated for all comparisons

**Split-half analysis result of seven-component model**

>> val7=splitvalidation(A1,7,[1 2;3 4;5 6],{'AB','CD','AC','BD','AD','BC'},LSmodel7r);

*Output:*

Overall Result = Not Validated

In the following tables, Match = 1, No Match = 0

Rows and columns are interpreted as follows:

Rows: depict the 1st model in each comparison

Columns: depict the 2nd model in each comparison

Model 7:AB vs CD-Not Validated

1 0 0 0 0 0 0

0 0 0 0 0 0 0

0 0 0 1 0 0 0

0 0 1 0 0 0 0

0 0 0 0 1 0 0

0 0 0 0 0 0 0

0 0 0 0 0 1 0

Model 7:AC vs BD-Validated

Model 7:AD vs BC-Validated

# **S5 Text. Fluorescence regional integration (FRI)**

The 130 data sets after collection and pre-processing including inner filter effects correction, Raman intensity normalization, subtraction of blank fluorescent spectra, multiplication by dilution factor, scatters removal, and interpolation were further analyzed by the FRI method. The EEM spectra of DOM were divided into two regions: region I represents soluble microbial by-product-like or tryptophan and protein-like compounds that are related to biological; region II represents a humic acid-like region [10]. The excitation/emission wavelength range of regions I and II were 250−450/280−380 nm and 250−450/380−550 nm respectively. The FRI technique was applied to integrate the area beneath EEM spectra. The volume (Φi) beneath region “*i*” of EEM can be calculated with the following equation [10, 11]:

$\Phi_{i}=\int_{ex}\int_{em}I\left( \lambda_{ex}\lambda_{em} \right)d\lambda_{ex}d\lambda_{em}$ (3)

For discrete data, the volumes (Φi) were expressed by:

$\Phi_{i}=\sum_{ex} \sum_{em} I (\lambda_{ex}\lambda_{em})\Delta\lambda_{ex}\Delta\lambda_{em}$ (4)

In which, Δλ_ex_, and Δλ_em_ are the excitation and emission increments (both were 5 nm), respectively, and *I* is the fluorescent intensity at each excitation-emission wavelength pair. The percentage of volumetric integration under each region was calculated with:

$\Phi_{T, 2}=\sum_{i=1}^{2} \Phi_{i, 2}$ (5)
$P_{i,2}= \frac{\Phi_{i, 2}}{\Phi_{T, 2}}\times100\%$ (6)

Φ_(T,2)_ was the total cumulative beneath EEM of 2 regions, P*_i,2_* was the percentage of the volume of region *i* (*n* = 2). The results of fluorescence regional integration are presented in S6 Table.

# **S6 Text. Comparison results between EEM-PARAFAC and FRI methods**

Region I showed a very strong positive correlation with the F_max_ value of C3 (*r* = 0.97, *p <* 0.001, *n* = 126), and region II showed a very strong correlation with F_max_ values of C1, C2, or C1+C2 (*r =* 0.96, *r =* 0.96, *r =* 1.00, all *p <* 0.001, respectively). Besides, a high positive correlation between region I and the F_max_ value of C1 (*r* = 0.94, *p <* 0.001) revealed the association between microbial-humic-like components (C1) with microbial activity or soluble microbial by-product-like components. This indicated the consistent results in assessing variations of protein-like components and humic-like components in landfill leachate by both FRI and EEM-PARAFAC methods. Furthermore, the BIX and the percentage of protein-like region I (%P1) demonstrated a positive correlation (*r* = 0.68, *p <* 0.001), which was consistent with the BIX and %C3 correlation (*r* = 0.52, *p* < 0.001) obtained from the PARAFAC analysis. The PARAFAC analysis revealed a significant negative correlation between %C3 and HIX (*r =* −0.92, *p* < 0.001) and the FRI revealed a significant negative correlation between %P1 and HIX (*r* = −0.94, *p* < 0.001). No strong positive correlation was observed between the humic-acid-like region and SUVA_254_ (*r =* −0.22, *p* < 0.05) or HIX (*r* = −0.43, *p* < 0.001). However, a strong positive correlation was observed between the percentage of the humic acid-like region II (%P2) and HIX (*r* = 0.94, *p* < 0.001), while a weak positive correlation was observed between the %P2 and SUVA_254_ (*r* = 0.25, *p* < 0.01). These findings from FRI and PARAFAC were in agreement that HIX could serve as an indicator for representing the percentage of fluorescent humic acid-like compounds in landfill leachate.

The results of the FRI method showed the percentage of microbial by-product-like or Tryptophan and protein-like compounds (region 1) ranged from 18% to 35% meanwhile humic acid-like compounds (region 2) ranged from 65.0% to 82% among all samples through 13 months (Fig. S8a). Region 1 exhibited the highest average percentage in G1.1 (32.7%) and G3 samples (29.9%), and the lowest average percentage in G4, G2, and G5 (23.0%, 22.9%, 23.1%). In contrast, the humic-acid region exhibited significant proportions in G4, G2, and G5 samples, accounting for 77.0%, 77.1%, and 76.9% of the total, respectively. The results indicated a lower proportion of fluorescence integration volume of the protein-like region compared with the humic-like fluorescence region. This trend was comparable with results of F_max_ C3 results of EEM-PARAFAC results (Fig. S8b), which indicated a higher total contribution of F_max_ of microbial and terrestrial humic-like peaks compared with protein-like peaks.

Cui et al. [12] stated that certain compounds present in the humic-like region (fluorescence intensity of hydrophobic acids and neutral organic fractions) are biodegradable and not refractory in nature. The FRI results showed a higher proportion of humic-like region compared with proportion F_max_ values of C1 + C2 and a lower proportion of protein-region compared with proportion F_max_ value of C3.

In the comparison of PARAFAC with FRI results in FDOM identification, the terrestrial humic-like component (C2) (Ex/Em <250, 350/450 nm) was determined to be associated with region 2 (humic-like region), and the tryptophan/ protein-like component (C3) (Ex/Em 280/345 nm) was found to be located in region 1 (protein-like region) meanwhile microbial humic-like component (C1) (Ex/Em <250, 320/395 nm) was located between region I and region II. It was observed that region I not only overlapped with C3, which decreased the humification degree but also overlapped with C1, which could increase the humification degree. While both EEM-PARAFAC and FRI are useful methods for roughly monitoring the variations of FDOM, EEM-PARAFAC demonstrated advantages in deconvoluting FDOM into distinct fluorescent components and mitigating peak overlap, emphasizing the significance of the characterization method. Furthermore, compared to C1, C2 has longer emission, which could be related to increasing carboxyl and hydroxyl groups, and have a higher degree of polycondensation [13]. Therefore, separating the humic-like components into C1 and C2 and monitoring their variations could be valuable when considering their aromaticity, degree of humification, functional groups, or interaction with pollutants or other environmental factors. The difference in characteristics of C1, C2, and C3 could lead to the difference in binding affinity with pollutants such as heavy metals [14, 15].

# **S1 Table. Explorative data analysis results and core consistency.**

| **Component no.** | **Iterations** | **Explained of residual  (%)** | **Core consistency  (%)** | **Sum of squared  of residuals** |
| --- | --- | --- | --- | --- |
| **2** | 20 | 97.95 | 94.06 | 11068.87 |
| **3** | 53 | 99.27 | 84.10 | 3914.70 |
| **4** | 60 | 99.53 | 2.92 | 2496.38 |
| **5** | 82 | 99.70 | 1.73 | 1639.89 |
| **6** | 190 | 99.85 | 2.74 | 814.77 |
| **7** | 214 | 99.88 | 1.28 | 631.40 |

#

# **S2 Table. Description of optical indices [27].**

| **Spectroscopic indices** | **Calculation** | **Purpose** | **References** |
| --- | --- | --- | --- |
| **Specific ultraviolet absorbance at 254 nm [SUVA_254_ (L/mg C·m)]** | Absorption coefficient at 254 nm (cm^–1^) divided by DOC concentration (mg C/L), and then multiply with 100 (cm/m) | Absorbance per unit carbon. Typically, a higher number is associated with greater aromatic content | [17] |
| **Flourescence index (FI)** | The ratio of Em wavelengths at 470 nm and 520 nm, obtained at Ex 370 nm | Shown to identify the relative contribution of  terrestrial and microbial sources to the DOM pool | [18, 19] |
| **Humification index (HIX)** | The area under the Em spectra 435–480 nm divided by the peak area 300–345 nm + 435–480 nm, at Ex 254 nm | An indicator of humic substance content or extent of humification. Higher values indicate an increasing degree of humification | [20] |
| **Biological index (BIX)** | The ratio of emission intensity at 380 nm  divided by 430 nm at Excitation 310 nm | An indicator of autotrophic productivity. High values  (> 1) correspond to recently produced DOM of autochthonous origin | [21] |
| **Freshness index (*β:α*)** | The ratio of Em intensity at 380 nm divided by the maximum emission intensity between 420 nm and 435 nm at excitation 310 nm | An indicator of recently produced DOM, with higher values representing a higher proportion of fresh DOM | [22, 23] |
| **Peak ratio (C:M)** | The ratio of Peak C (ex340/em440) to Peak M(ex300/em390) intensity | An indication of the amount of diagenetically altered (blue shifted) fluorescence in a sample | [24, 25] |
| **Peak ratio (C:T)** | The ratio of Peak C (ex340/em440) to Peak T (ex275/em304) intensity | An indication of the amount of humic-like(recalcitrant) vs. fresh-like (labile) fluorescence in a sample | [26] |
| **Peak ratio (C:A)** | The ratio of Peak C (ex340/em440) to Peak A (ex260/em450) intensity | An indication of the amount of humic-like vs. fulvic-like fluorescence in a sample | [19, 26] |
| **Peak ratio (A:T)** | The ratio of Peak A (ex260/em450) to Peak T (ex275/em304) intensity | An indication of the amount of humic-like (recalcitrant) vs. fresh-like (labile) fluorescence in a sample | [16] |
| **Peak ratio (M:T)** | The ratio of Peak M (ex300/em390) to Peak T(ex275/em304) intensity | An indication of the amount of microbial humic-like vs. fresh-like (labile) fluorescence in a sample | This study |

# **S3 Table. PARAFAC-derived components in this research compared to previous studies on the online library [20].**

| **Tucker congruence coefficients (TCC)** | **C1**  **(Number of comparable studies)**  **75 matched results** | **C2**  **(Number of comparable studies)**  **89 matched results** | **C3**  **(Number of comparable studies)**  **52 matched results** |
| --- | --- | --- | --- |
| **> 0.95**  **Excitation** | 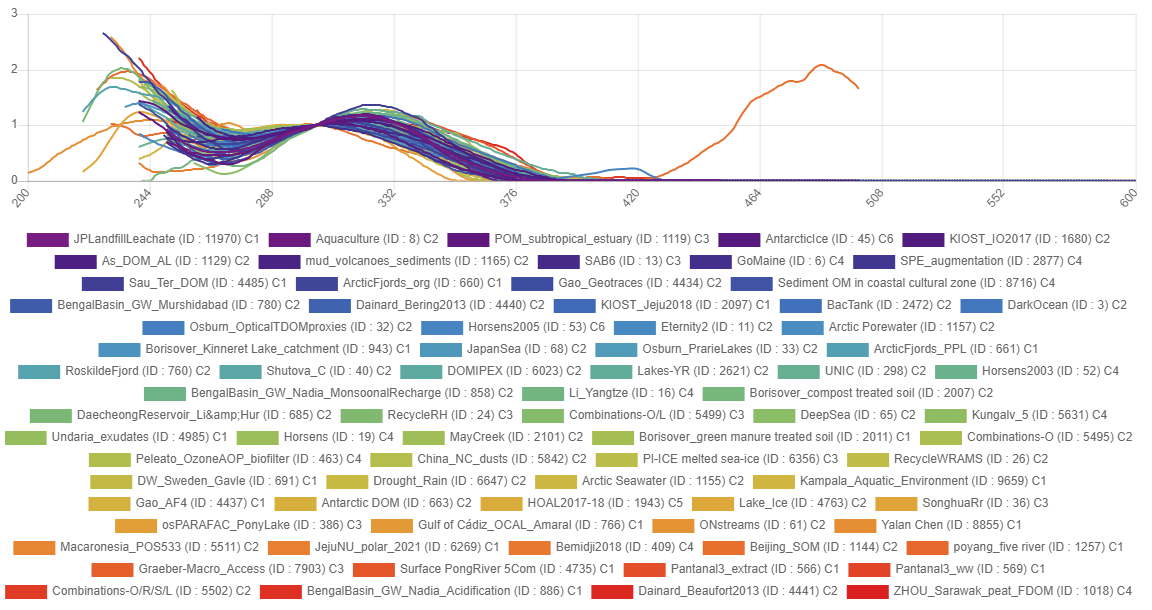 | 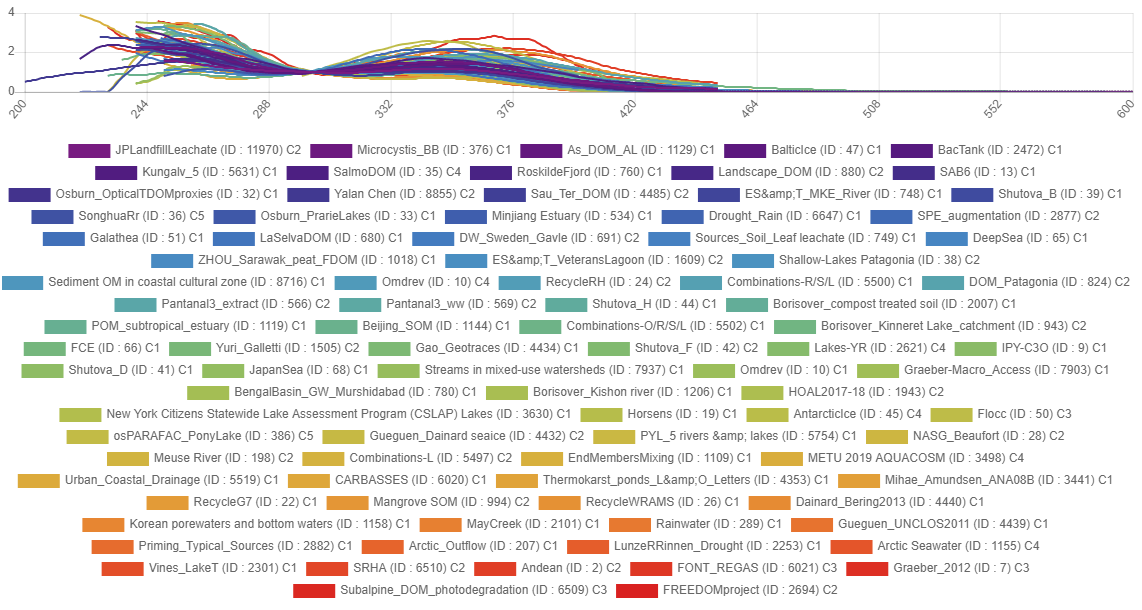 | 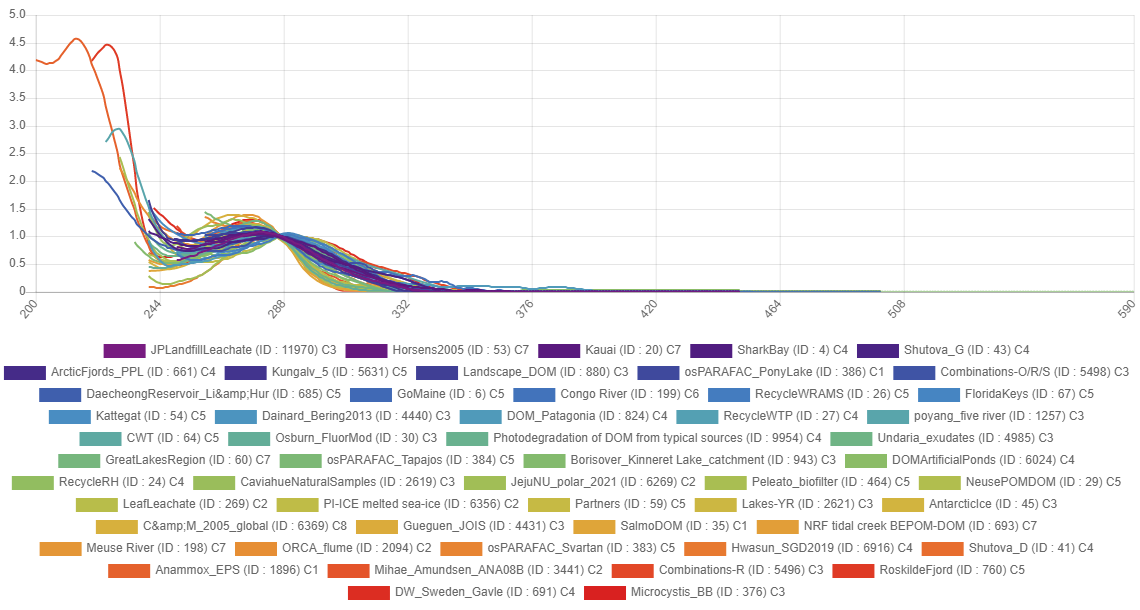 |
| **> 0.95**  **Emission** | 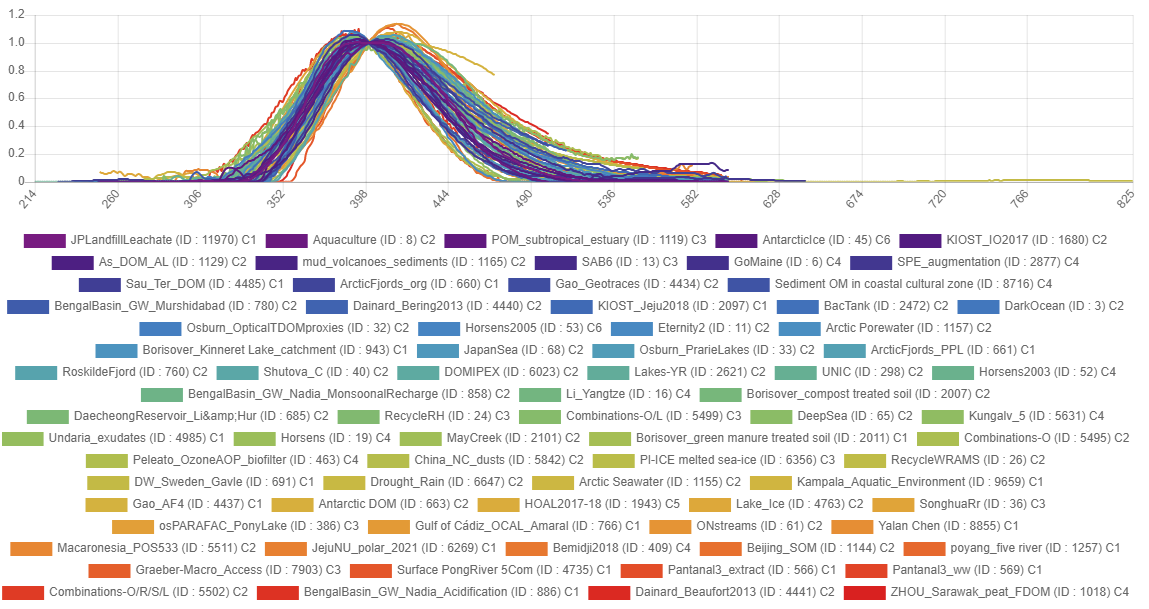 | 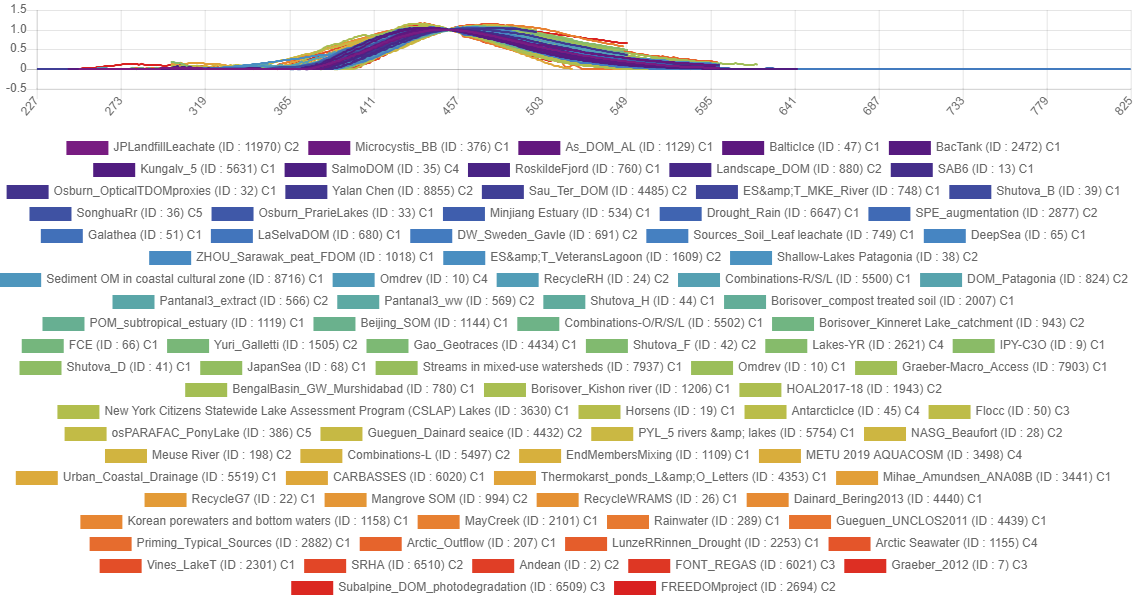 | 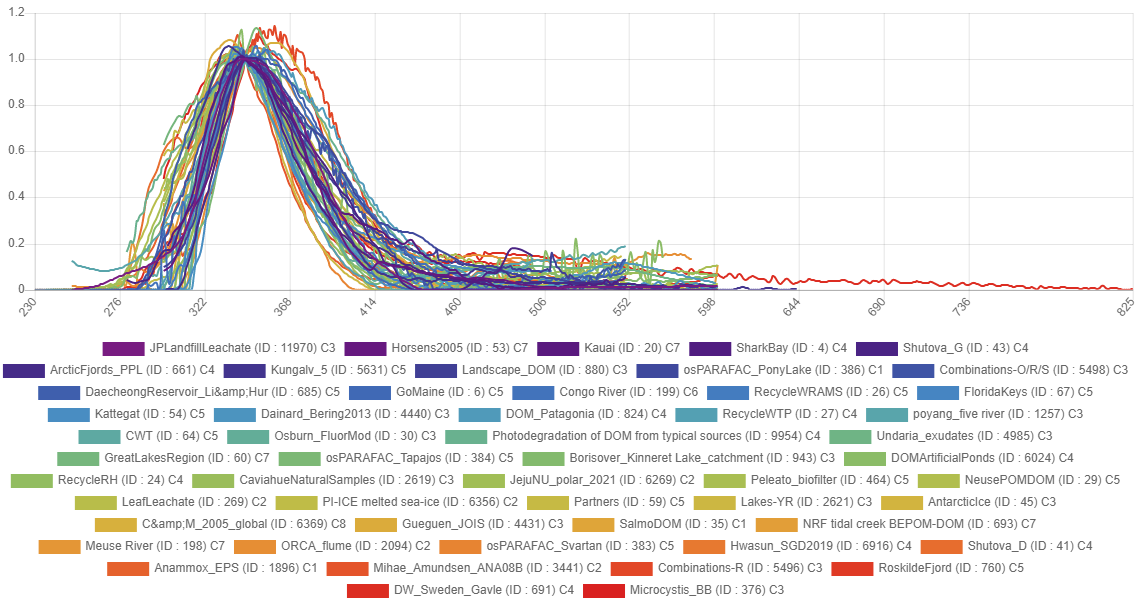 |
| **Sources** | Aquaculture, catchments, creek, estuaries, groundwater, headwaters, Japan seawater, lagoon, lakes, mud volcano, oceans, peat, rivers, sediments, streams, wetland | Aquaculture, catchment, estuaries, greywater, groundwater, lagoon, lakes, leaf leachate, oceans, peat, rivers, seawater, sediments, soil, soil leachate, streams, wastewater, wetland | Algae, artificial ponds, catchment, estuaries, greywater, groundwater, lakes, Microcystis, oceans, plant and leaf litter leachates, rivers, sea-ice, sewage, streams, urban ponds, wastewater |

# **S4 Table. The maximum fluorescence intensity of PARAFAC-derived components, DOC concentration, and spectroscopic indices.**

Min.–Max. (mean ± stdev), (*n* = 126), period: from *June 2019–June 2020*

| **Sampling sites** | **DOC conc.** | **F_max_ of C1** | **F_max_ of C2** | **F_max_ of C3** | **UVA_254_** | **SUVA_254_** | **HIX** | **FI** | **BIX** |
| --- | --- | --- | --- | --- | --- | --- | --- | --- | --- |
| **G1.1** | 10.7–19.0  (12.6 ± 2.0) | 36.5–60.8  (42.3 ± 7.0) | 27.5–41.0  (31.3 ± 3.8) | 46.1–61.9  (55.6 ± 5.1) | 0.26–0.30  (0.29 ± 0.01) | 1.38–2.69  (2.30 ± 0.31) | 0.67–0.74  (0.69 ± 0.02) | 2.73–3.04  (2.82 ± 0.09) | 1.02–1.05  (1.03 ± 0.01) |
| **G1.2** | 6.4–38.2  (15.3 ± 8.2) | 29.4–129.4  (54.2 ± 25.8) | 26.3–100.3  (43.9 ± 18.9) | 17.5–88.9  (40.3 ± 22.1) | 0.19–0.74  (0.38 ± 0.14) | 1.92–3.65  (2.67 ± 0.48) | 0.72–0.87  (0.79 ± 0.05) | 2.67–3.03  (2.84 ± 0.12) | 0.90–1.04  (0.98 ± 0.04) |
| **G2** | 5.9–15.6  (10.0 ± 2.7) | 18.3–51.5  (29.4 ± 9.2) | 16.0–47.2  (25.2 ± 8.4) | 10.7–30.6  (19.0 ± 5.7) | 0.16–0.40  (0.26 ± 0.08) | 2.04–4.74  (2.61 ± 0.71) | 0.79–0.83  (0.81 ± 0.01) | 2.55–2.76  (2.64 ± 0.06) | 0.87–0.93  (0.90 ± 0.02) |
| **G3** | 13.5–20.8  (17.8 ± 2.1) | 46.6–98.5  (72.0 ± 15.4) | 34.4–51.5  (41.4 ± 5.2) | 29.1–88.0  (65.3 ± 18.8) | 0.46–0.79  (0.60 ± 0.12) | 2.62–4.81  (3.40 ± 0.77) | 0.61–0.82  (0.69 ± 0.06) | 2.63–3.33  (3.12 ± 0.19) | 0.97–1.26  (1.09 ± 0.08) |
| **G4** | 9.2–18.9  (13.2 ± 2.7) | 19.9–40.1  (29.1 ± 6.1) | 26.1–41.6  (34.3 ± 4.9) | 15.5–40.7  (26.7 ± 8.4) | 0.44–0.71  (0.55 ± 0.10) | 2.56–4.90  (4.24 ± 0.60) | 0.73–0.85  (0.80 ± 0.04) | 2.10–2.97  (2.30 ± 0.14) | 0.71–1.05  (0.81 ± 0.10) |
| **G5** | 2.6–8.0  (5.6 ± 1.8) | 3.6–27.3  (15.0 ± 8.4) | 3.9–21.7  (12.7 ± 6.3) | 2.0–23.6  (10.7 ± 7.0) | 0.10–0.31  (0.21 ± 0.07) | 2.42–5.77  (3.99 ± 1.22) | 0.76–0.88  (0.80 ± 0.03) | 2.37–2.82  (2.64 ± 0.14) | 0.85–0.94  (0.91 ± 0.03) |
| **L1** | 2.7–15.8  (12.4 ± 4.0) | 9.4–52.1  (39.1 ± 13.0) | 8.2–29.9  (22.6 ± 6.3) | 4.0–37.2  (27.7 ± 10.2) | 0.07–0.38  (0.27 ± 0.09) | 1.76–2.65  (2.20 ± 0.25) | 0.72–0.87  (0.74 ± 0.04) | 2.60–3.06  (2.96 ± 0.13) | 0.87–1.05  (1.00 ± 0.04) |
| **L2** | 6.4–10.4  (8.3 ± 1.1) | 21.5–42.2  (33.1 ± 5.7) | 16.6–35.3  (26.1 ± 4.9) | 12.5–37.0  (25.2 ± 6.2) | 0.15–0.22  (0.19 ± 0.02) | 2.01–2.84  (2.26 ± 0.21) | 0.72–0.81  (0.77 ± 0.03) | 2.75–3.06  (2.89 ± 0.09) | 0.61–0.99  (0.79 ± 0.13) |
| **L3** | 6.5–11.4  (9.3 ± 1.2) | 22.7–52.0  (36.9 ± 6.7) | 18.1–35.4  (26.4 ± 4.1) | 13.8–36.8  (22.1 ± 5.4) | 0.11–0.26  (0.19 ± 0.04) | 1.02–2.44  (2.10 ± 0.35) | 0.75–0.81  (0.79 ± 0.02) | 2.81–3.10  (2.88 ± 0.08) | 0.74–1.00  (0.88 ± 0.08) |
| **L4** | 6.1–11.5  (8.6 ± 1.3) | 21.1–39.2  (33.8 ± 4.9) | 17.9–29.1  (24.5 ± 3.2) | 16.0–28.1  (20.2 ± 3.3) | 0.14–0.23  (0.19 ± 0.02) | 1.61–2.55  (2.24 ± 0.25) | 0.75–0.82  (0.79 ± 0.02) | 2.78–3.20  (2.90 ± 0.10) | 0.73–1.00  (0.86 ± 0.09) |

# **S5 Table. Leachate level, F_max_ of PARAFAC-derived components, and DOC concentration**

| **ID** | **Leachate level (m)** | **F_max_ of C1 (RU)** | **F_max_ of C2 (RU)** | **F_max_ of C3 (RU)** | **DOC conc.  (mg C/ L)** |
| --- | --- | --- | --- | --- | --- |
| 01G1-1 | 6.7 | 53.3 | 37.0 | 46.1 | 19.0 |
| 02G1-1 | 7.3 | 60.8 | 41.0 | 56.7 | 12.9 |
| 03G1-1 | 6.8 | 36.5 | 29.3 | 53.3 | 13.2 |
| 04G1-1 | 6.7 | 36.6 | 28.8 | 59.0 | 11.9 |
| 05G1-1 | 6.9 | 39.8 | 31.2 | 61.2 | 12.3 |
| 07G1-1 | 6.9 | 41.4 | 31.2 | 59.5 | 12.0 |
| 08G1-1 | 7.2 | 39.6 | 27.9 | 49.4 | 12.0 |
| 09G1-1 | 7.2 | 40.4 | 27.5 | 48.1 | 12.0 |
| 10G1-1 | 7.0 | 43.6 | 31.5 | 55.6 | 12.5 |
| 11G1-1 | 6.9 | 41.6 | 31.4 | 59.1 | 10.7 |
| 12G1-1 | 7.2 | 39.0 | 30.6 | 61.9 | 11.9 |
| 13G1-1 | 7.0 | 39.3 | 29.6 | 57.3 | 11.3 |
| 01G1-2 | 11.4 | 129.4 | 100.3 | 88.9 | 38.2 |
| 02G1-2 | 10.6 | 48.9 | 43.0 | 28.9 | 14.8 |
| 03G1-2 | 10.1 | 40.2 | 38.3 | 19.6 | 9.2 |
| 04G1-2 | 10.1 | 40.6 | 35.8 | 22.6 | 10.1 |
| 05G1-2 | 10.2 | 58.6 | 45.6 | 46.2 | 15.6 |
| 07G1-2 | 9.1 | 71.8 | 50.7 | 63.5 | 21.9 |
| 08G1-2 | 10.5 | 36.3 | 28.1 | 26.4 | 9.5 |
| 09G1-2 | 10.6 | 40.2 | 32.8 | 28.6 | 11.8 |
| 10G1-2 | 9.5 | 43.9 | 39.5 | 28.2 | 11.9 |
| 11G1-2 | 10.4 | 29.4 | 26.3 | 17.5 | 6.4 |
| 12G1-2 | 10.6 | 63.6 | 50.2 | 62.0 | 19.5 |
| 13G1-2 | 10.7 | 62.1 | 49.6 | 62.1 | 17.0 |
| 01G2 | 6.1 | 51.5 | 47.2 | 30.6 | 15.6 |
| 02G2 | 5.5 | 39.0 | 31.6 | 22.0 | 14.0 |
| 03G2 | 4.9 | 22.5 | 18.7 | 14.4 | 8.7 |
| 04G2 | 5.0 | 19.3 | 16.0 | 13.7 | 7.2 |
| 05G2 | 5.1 | 26.5 | 22.5 | 17.1 | 9.3 |
| 06G2 | 5.0 | 21.5 | 18.0 | 14.3 | 7.8 |
| 07G2 | 5.7 | 27.6 | 23.0 | 18.0 | 9.7 |
| 08G2 | 5.6 | 31.1 | 25.5 | 19.7 | 10.4 |
| 09G2 | 5.5 | 29.0 | 24.5 | 19.2 | 12.1 |
| 10G2 | 5.4 | 36.5 | 32.2 | 28.3 | 11.6 |
| 11G2 | 5.0 | 33.3 | 28.5 | 22.7 | 9.2 |
| 12G2 | 5.2 | 25.6 | 22.8 | 16.5 | 8.6 |
| 13G2 | 5.2 | 18.3 | 16.8 | 10.7 | 5.9 |

**S5 Table. (*continued*)**

| **ID** | **Leachate level (m)** | **F_max_ of C1 (RU)** | **F_max_ of C2 (RU)** | **F_max_ of C3 (RU)** | **DOC conc.  (mg C/ L)** |
| --- | --- | --- | --- | --- | --- |
| 01G3 | 9.9 | 90.5 | 40.0 | 43.8 | 18.8 |
| 02G3 | 9.8 | 46.6 | 41.5 | 29.1 | 16.4 |
| 07G3 | 8.8 | 98.5 | 51.5 | 81.2 | 18.9 |
| 08G3 | 9.0 | 71.7 | 40.5 | 59.5 | 18.1 |
| 09G3 | 9.0 | 70.6 | 40.2 | 71.2 | 18.9 |
| 10G3 | 8.8 | 76.2 | 43.2 | 88.0 | 20.8 |
| 11G3 | 8.8 | 67.3 | 40.4 | 84.5 | 16.6 |
| 12G3 | 9.0 | 63.6 | 34.7 | 64.5 | 17.0 |
| 13G3 | 9.9 | 55.2 | 34.4 | 55.5 | 13.5 |
| 01G4 | 7.4 | 34.3 | 34.4 | 39.9 | 14.0 |
| 02G4 | 6.6 | 40.1 | 26.1 | 39.4 | 10.6 |
| 03G4 | 6.1 | 35.8 | 40.1 | 40.7 | 18.9 |
| 04G4 | 6.2 | 30.2 | 38.5 | 26.3 | 16.7 |
| 05G4 | 6.7 | 31.2 | 39.9 | 25.6 | 15.4 |
| 06G4 | 6.2 | 31.5 | 41.6 | 27.0 | 14.5 |
| 07G4 | 6.4 | 34.2 | 27.9 | 25.7 | 14.4 |
| 08G4 | 6.6 | 26.3 | 35.5 | 20.5 | 12.3 |
| 09G4 | 6.6 | 24.1 | 33.7 | 19.3 | 11.8 |
| 10G4 | 6.5 | 24.9 | 34.1 | 24.3 | 11.3 |
| 11G4 | 6.3 | 19.9 | 28.7 | 17.9 | 9.2 |
| 12G4 | 7.6 | 25.6 | 34.3 | 25.4 | 11.4 |
| 13G4 | 6.8 | 20.6 | 31.5 | 15.5 | 11.0 |
| 01G5 | 7.6 | 24.5 | 21.5 | 17.1 | 7.3 |
| 02G5 | 7.4 | 6.4 | 6.1 | 3.4 | 3.7 |
| 03G5 | 7.0 | 6.9 | 6.4 | 4.3 | 4.1 |
| 04G5 | 7.1 | 12.3 | 10.8 | 8.3 | 4.8 |
| 05G5 | 7.3 | 18.4 | 15.6 | 12.4 | 6.6 |
| 06G5 | 6.8 | 7.8 | 7.3 | 4.9 | 5.6 |
| 07G5 | 7.3 | 10.0 | 9.5 | 6.3 | 4.8 |
| 08G5 | 6.8 | 21.5 | 17.3 | 19.2 | 7.0 |
| 09G5 | 7.5 | 27.3 | 21.7 | 23.6 | 7.8 |
| 10G5 | 7.5 | 25.0 | 19.1 | 16.4 | 8.0 |
| 11G5 | 7.7 | 3.6 | 3.9 | 2.0 | 2.6 |
| 12G5 | 7.8 | 23.0 | 17.5 | 15.2 | 6.9 |
| 13G5 | 7.8 | 8.8 | 8.1 | 5.4 | 3.7 |

# **S6 Table. Results of fluorescence regional integration**

| **Sampling**  **site** | **ID** | **Region 1 (RU)** | **Region 2 (RU)** | **Total regions (RU)** | **P1 (%)** | **P2 (%)** |
| --- | --- | --- | --- | --- | --- | --- |
| G1-1 | 01G1-1 | 2.27E+05 | 5.80E+05 | 8.07E+05 | 28.09 | 71.91 |
|  | 02G1-1 | 2.70E+05 | 6.67E+05 | 9.37E+05 | 28.83 | 71.17 |
|  | 03G1-1 | 2.15E+05 | 4.33E+05 | 6.48E+05 | 33.15 | 66.85 |
|  | 04G1-1 | 2.30E+05 | 4.28E+05 | 6.59E+05 | 34.98 | 65.02 |
|  | 05G1-1 | 2.41E+05 | 4.62E+05 | 7.04E+05 | 34.29 | 65.71 |
|  | 06G1-1 | 2.26E+05 | 4.44E+05 | 6.70E+05 | 33.74 | 66.26 |
|  | 07G1-1 | 2.39E+05 | 4.71E+05 | 7.11E+05 | 33.70 | 66.30 |
|  | 08G1-1 | 2.09E+05 | 4.38E+05 | 6.48E+05 | 32.32 | 67.68 |
|  | 09G1-1 | 2.07E+05 | 4.38E+05 | 6.46E+05 | 32.11 | 67.89 |
|  | 10G1-1 | 2.35E+05 | 4.88E+05 | 7.23E+05 | 32.50 | 67.50 |
|  | 11G1-1 | 2.40E+05 | 4.77E+05 | 7.17E+05 | 33.48 | 66.52 |
|  | 12G1-1 | 2.42E+05 | 4.56E+05 | 6.99E+05 | 34.68 | 65.32 |
|  | 13G1-1 | 2.30E+05 | 4.52E+05 | 6.82E+05 | 33.75 | 66.25 |
| G1-2 | 01G1-2 | 5.09E+05 | 1.56E+06 | 2.07E+06 | 24.63 | 75.37 |
|  | 02G1-2 | 1.76E+05 | 6.41E+05 | 8.17E+05 | 21.55 | 78.45 |
|  | 03G1-2 | 1.29E+05 | 5.35E+05 | 6.64E+05 | 19.39 | 80.61 |
|  | 04G1-2 | 1.44E+05 | 5.25E+05 | 6.68E+05 | 21.48 | 78.52 |
|  | 05G1-2 | 2.44E+05 | 7.08E+05 | 9.51E+05 | 25.60 | 74.40 |
|  | 06G1-2 | 1.70E+05 | 4.85E+05 | 6.56E+05 | 25.99 | 74.01 |
|  | 07G1-2 | 3.23E+05 | 8.32E+05 | 1.15E+06 | 27.97 | 72.03 |
|  | 08G1-2 | 1.43E+05 | 4.32E+05 | 5.75E+05 | 24.86 | 75.14 |
|  | 09G1-2 | 1.57E+05 | 4.93E+05 | 6.51E+05 | 24.16 | 75.84 |
|  | 10G1-2 | 1.64E+05 | 5.72E+05 | 7.37E+05 | 22.31 | 77.69 |
|  | 11G1-2 | 1.06E+05 | 3.81E+05 | 4.88E+05 | 21.82 | 78.18 |
|  | 12G1-2 | 2.98E+05 | 7.92E+05 | 1.09E+06 | 27.32 | 72.68 |
|  | 13G1-2 | 2.94E+05 | 7.81E+05 | 1.07E+06 | 27.32 | 72.68 |
| G2 | 01G2 | 1.87E+05 | 6.60E+05 | 8.47E+05 | 22.04 | 77.96 |
|  | 02G2 | 1.34E+05 | 4.79E+05 | 6.14E+05 | 21.89 | 78.11 |
|  | 03G2 | 8.12E+04 | 2.69E+05 | 3.50E+05 | 23.19 | 76.81 |
|  | 04G2 | 7.28E+04 | 2.23E+05 | 2.95E+05 | 24.64 | 75.36 |
|  | 05G2 | 9.19E+04 | 3.04E+05 | 3.96E+05 | 23.21 | 76.79 |
|  | 06G2 | 7.69E+04 | 2.49E+05 | 3.26E+05 | 23.60 | 76.40 |
|  | 07G2 | 9.59E+04 | 3.11E+05 | 4.07E+05 | 23.54 | 76.46 |
|  | 08G2 | 1.10E+05 | 3.63E+05 | 4.73E+05 | 23.18 | 76.82 |
|  | 09G2 | 1.06E+05 | 3.51E+05 | 4.58E+05 | 23.21 | 76.79 |
|  | 10G2 | 1.44E+05 | 4.49E+05 | 5.93E+05 | 24.30 | 75.70 |
|  | 11G2 | 1.24E+05 | 4.02E+05 | 5.26E+05 | 23.57 | 76.43 |
|  | 12G2 | 8.98E+04 | 3.07E+05 | 3.96E+05 | 22.66 | 77.34 |
|  | 13G2 | 6.08E+04 | 2.23E+05 | 2.84E+05 | 21.45 | 78.55 |

**S6 Table. *(continued).***

| **Sampling site** | **ID** | **Region 1 (RU)** | **Region 2 (RU)** | **Total regions (RU)** | **P1 (%)** | **P2 (%)** |
| --- | --- | --- | --- | --- | --- | --- |
| G3 | 01G3 | 2.99E+05 | 8.14E+05 | 1.11E+06 | 26.89 | 73.11 |
|  | 02G3 | 1.74E+05 | 6.10E+05 | 7.84E+05 | 22.18 | 77.82 |
|  | 06G3 | 3.67E+05 | 8.57E+05 | 1.22E+06 | 29.96 | 70.04 |
|  | 07G3 | 4.32E+05 | 9.97E+05 | 1.43E+06 | 30.23 | 69.77 |
|  | 08G3 | 3.05E+05 | 7.45E+05 | 1.05E+06 | 29.04 | 70.96 |
|  | 09G3 | 3.36E+05 | 7.40E+05 | 1.08E+06 | 31.25 | 68.75 |
|  | 10G3 | 3.93E+05 | 8.06E+05 | 1.20E+06 | 32.81 | 67.19 |
|  | 11G3 | 3.87E+05 | 7.52E+05 | 1.14E+06 | 33.98 | 66.02 |
|  | 12G3 | 3.02E+05 | 6.57E+05 | 9.59E+05 | 31.49 | 68.51 |
|  | 13G3 | 2.69E+05 | 6.02E+05 | 8.71E+05 | 30.94 | 69.06 |
| G4 | 01G4 | 1.79E+05 | 4.60E+05 | 6.39E+05 | 27.99 | 72.01 |
|  | 02G4 | 1.84E+05 | 4.43E+05 | 6.27E+05 | 29.31 | 70.69 |
|  | 03G4 | 1.91E+05 | 5.54E+05 | 7.45E+05 | 25.58 | 74.42 |
|  | 04G4 | 1.33E+05 | 4.76E+05 | 6.08E+05 | 21.82 | 78.18 |
|  | 05G4 | 1.32E+05 | 4.94E+05 | 6.26E+05 | 21.04 | 78.96 |
|  | 06G4 | 1.43E+05 | 5.17E+05 | 6.60E+05 | 21.67 | 78.33 |
|  | 07G4 | 1.29E+05 | 3.66E+05 | 4.95E+05 | 26.01 | 73.99 |
|  | 08G4 | 1.05E+05 | 4.29E+05 | 5.34E+05 | 19.74 | 80.26 |
|  | 09G4 | 1.29E+05 | 3.66E+05 | 4.95E+05 | 26.01 | 73.99 |
|  | 10G4 | 9.82E+04 | 4.06E+05 | 5.04E+05 | 19.49 | 80.51 |
|  | 11G4 | 8.74E+04 | 3.43E+05 | 4.30E+05 | 20.31 | 79.69 |
|  | 12G4 | 1.22E+05 | 4.18E+05 | 5.40E+05 | 22.52 | 77.48 |
|  | 13G4 | 8.14E+04 | 3.71E+05 | 4.53E+05 | 17.97 | 82.03 |
| G5 | 01G5 | 9.36E+04 | 3.00E+05 | 3.93E+05 | 23.79 | 76.21 |
|  | 02G5 | 2.09E+04 | 8.49E+04 | 1.06E+05 | 19.77 | 80.23 |
|  | 03G5 | 2.46E+04 | 8.67E+04 | 1.11E+05 | 22.09 | 77.91 |
|  | 04G5 | 4.55E+04 | 1.51E+05 | 1.96E+05 | 23.17 | 76.83 |
|  | 05G5 | 6.72E+04 | 2.20E+05 | 2.87E+05 | 23.41 | 76.59 |
|  | 06G5 | 2.78E+04 | 9.83E+04 | 1.26E+05 | 22.03 | 77.97 |
|  | 07G5 | 3.54E+04 | 1.26E+05 | 1.62E+05 | 21.86 | 78.14 |
|  | 08G5 | 9.17E+04 | 2.54E+05 | 3.46E+05 | 26.50 | 73.50 |
|  | 09G5 | 9.04E+04 | 2.87E+05 | 3.78E+05 | 23.93 | 76.07 |
|  | 10G5 | 1.14E+05 | 3.22E+05 | 4.36E+05 | 26.11 | 73.89 |
|  | 11G5 | 1.21E+04 | 4.95E+04 | 6.17E+04 | 19.69 | 80.31 |
|  | 12G5 | 2.70E+05 | 6.67E+05 | 9.37E+05 | 28.83 | 71.17 |
|  | 13G5 | 3.07E+04 | 1.11E+05 | 1.41E+05 | 21.69 | 78.31 |

**S6 Table. *(continued).***

| **Sampling site** | **ID** | **Region 1 (RU)** | **Region 2 (RU)** | **Total regions (RU)** | **P1 (%)** | **P2 (%)** |
| --- | --- | --- | --- | --- | --- | --- |
| L1 | 01L1 | 2.06E+05 | 5.19E+05 | 7.25E+05 | 28.36 | 71.64 |
|  | 02L1 | 1.48E+05 | 3.96E+05 | 5.44E+05 | 27.23 | 72.77 |
|  | 04L1 | 1.45E+05 | 3.90E+05 | 5.35E+05 | 27.15 | 72.85 |
|  | 05L1 | 1.62E+05 | 4.24E+05 | 5.86E+05 | 27.59 | 72.41 |
|  | 06L1 | 2.70E+05 | 6.67E+05 | 9.37E+05 | 28.83 | 71.17 |
|  | 07L1 | 1.76E+05 | 4.52E+05 | 6.28E+05 | 28.00 | 72.00 |
|  | 08L1 | 1.94E+05 | 4.92E+05 | 6.86E+05 | 28.29 | 71.71 |
|  | 09L1 | 1.81E+05 | 4.61E+05 | 6.42E+05 | 28.19 | 71.81 |
|  | 10L1 | 1.80E+05 | 4.57E+05 | 6.37E+05 | 28.22 | 71.78 |
|  | 11L1 | 2.75E+04 | 1.14E+05 | 1.41E+05 | 19.48 | 80.52 |
|  | 12L1 | 1.58E+05 | 4.03E+05 | 5.61E+05 | 28.13 | 71.87 |
|  | 13L1 | 1.80E+05 | 4.66E+05 | 6.46E+05 | 27.85 | 72.15 |
| L2 | 01L2 | 1.39E+05 | 4.12E+05 | 5.51E+05 | 25.22 | 74.78 |
|  | 02L2 | 1.42E+05 | 3.73E+05 | 5.15E+05 | 27.54 | 72.46 |
|  | 03L2 | 7.42E+04 | 2.48E+05 | 3.22E+05 | 23.03 | 76.97 |
|  | 04L2 | 1.01E+05 | 3.44E+05 | 4.45E+05 | 22.70 | 77.30 |
|  | 05L2 | 1.15E+05 | 3.97E+05 | 5.12E+05 | 22.47 | 77.53 |
|  | 06L2 | 1.08E+05 | 3.95E+05 | 5.04E+05 | 21.50 | 78.50 |
|  | 07L2 | 1.44E+05 | 5.08E+05 | 6.52E+05 | 22.07 | 77.93 |
|  | 08L2 | 1.30E+05 | 3.92E+05 | 5.21E+05 | 24.91 | 75.09 |
|  | 09L2 | 1.81E+05 | 5.18E+05 | 6.99E+05 | 25.88 | 74.12 |
|  | 10L2 | 1.52E+05 | 4.04E+05 | 5.56E+05 | 27.40 | 72.60 |
|  | 11L2 | 1.48E+05 | 3.99E+05 | 5.47E+05 | 27.04 | 72.96 |
|  | 12L2 | 1.42E+05 | 3.81E+05 | 5.23E+05 | 27.23 | 72.77 |
|  | 13L2 | 1.32E+05 | 3.71E+05 | 5.03E+05 | 26.23 | 73.77 |
| L3 | 01L3 | 1.74E+05 | 5.26E+05 | 7.00E+05 | 24.88 | 75.12 |
|  | 02L3 | 1.29E+05 | 3.94E+05 | 5.23E+05 | 24.60 | 75.40 |
|  | 03L3 | 8.01E+04 | 2.67E+05 | 3.47E+05 | 23.05 | 76.95 |
|  | 04L3 | 1.07E+05 | 3.75E+05 | 4.82E+05 | 22.15 | 77.85 |
|  | 05L3 | 1.16E+05 | 3.99E+05 | 5.16E+05 | 22.57 | 77.43 |
|  | 06L3 | 1.11E+05 | 3.91E+05 | 5.02E+05 | 22.09 | 77.91 |
|  | 07L3 | 1.30E+05 | 4.49E+05 | 5.79E+05 | 22.53 | 77.47 |
|  | 08L3 | 1.27E+05 | 4.03E+05 | 5.30E+05 | 23.99 | 76.01 |
|  | 09L3 | 1.81E+05 | 5.24E+05 | 7.04E+05 | 25.65 | 74.35 |
|  | 10L3 | 1.28E+05 | 4.01E+05 | 5.29E+05 | 24.26 | 75.74 |
|  | 11L3 | 1.27E+05 | 3.85E+05 | 5.12E+05 | 24.80 | 75.20 |
|  | 12L3 | 1.38E+05 | 4.26E+05 | 5.64E+05 | 24.42 | 75.58 |
|  | 13L3 | 1.37E+05 | 4.42E+05 | 5.79E+05 | 23.72 | 76.28 |

**S6 Table. *(continued).***

| **Sampling site** | **ID** | **Region 1 (RU)** | **Region 2 (RU)** | **Total regions (RU)** | **P1 (%)** | **P2 (%)** |
| --- | --- | --- | --- | --- | --- | --- |
| **L4** | 01L4 | 1.46E+05 | 4.17E+05 | 5.63E+05 | 25.99 | 74.01 |
|  | 02L4 | 1.11E+05 | 3.36E+05 | 4.47E+05 | 24.83 | 75.17 |
|  | 03L4 | 8.33E+04 | 2.61E+05 | 3.45E+05 | 24.18 | 75.82 |
|  | 04L4 | 9.65E+04 | 3.37E+05 | 4.33E+05 | 22.28 | 77.72 |
|  | 05L4 | 1.07E+05 | 3.78E+05 | 4.85E+05 | 22.16 | 77.84 |
|  | 06L4 | 1.09E+05 | 3.94E+05 | 5.02E+05 | 21.67 | 78.33 |
|  | 07L4 | 1.26E+05 | 4.41E+05 | 5.68E+05 | 22.25 | 77.75 |
|  | 08L4 | 1.20E+05 | 3.89E+05 | 5.09E+05 | 23.67 | 76.33 |
|  | 09L4 | 1.38E+05 | 4.38E+05 | 5.76E+05 | 23.96 | 76.04 |
|  | 10L4 | 1.20E+05 | 3.81E+05 | 5.01E+05 | 23.94 | 76.06 |
|  | 11L4 | 1.21E+05 | 3.77E+05 | 4.98E+05 | 24.35 | 75.65 |
|  | 12L4 | 1.28E+05 | 4.07E+05 | 5.34E+05 | 23.92 | 76.08 |
|  | 13L4 | 1.30E+05 | 4.15E+05 | 5.45E+05 | 23.80 | 76.20 |

# **S1 Fig.** **Schematic of procedures involved in PARAFAC analysis of EEMs.**

Modified from Murphy et al. [15]; Stedmon and Bro [6]

# **S2 Fig. The residual analysis of two- to seven-component models**

# **S3 Fig. The core consistency, variance explained, and component size of two- to seven-component models**

# **S4 Fig. Two fluorescence components identified by EEM– PARAFAC analysis and results of the split-half analysis.**

# **S5 Fig. Three fluorescence components identified by EEM– PARAFAC analysis and results of the split-half analysis.**

# **S6 Fig. Four fluorescence components identified by EEM– PARAFAC analysis and results of the split-half analysis.**

# **S7 Fig. Five fluorescence components identified by EEM– PARAFAC analysis and results of the split-half analysis.**

# **S8 Fig. Six fluorescence components identified by EEM– PARAFAC analysis and results of the split-half analysis.**

# **S9 Fig. Seven fluorescence components identified by EEM– PARAFAC analysis and results of the split-half analysis.**

# **S10 Fig.** Correlations between: (a) F_max_ of C1 and DOC concentration, (b) F_max_ of C2 and DOC concentration, (c) F_max_ of C3 and DOC concentration, (d) UVA_254_ and DOC concentration

# **S11 Fig. Relationship between F_max_ of C1 and DOC conc.** (*red eq: R^2^ > 0.6, black eq: R^2^ < 0.6*)

# **S12 Fig. Relationship between F_max_ of C2 and DOC conc.** (*red eq: R^2^ > 0.6, black eq: R^2^ < 0.6*)

# **S13 Fig. Relationship between F_max_ of C3 and DOC conc.** (*red eq: R^2^ > 0.6, black eq: R^2^ < 0.6*)

# **S14 Fig. Relationship between UVA_254_ and DOC conc.** (*red eq: R^2^ > 0.6, black eq: R^2^ < 0.6*)

# **S15 Fig. Correlations between PARAFAC–derived components and UVA_254_.** (a) F_max_ of C1 and UVA_254_, (b) F_max_ of C1 and UVA_254_, (c) F_max_ of C1 and UVA_254_

# **S16 Fig.** Correlations between **PARAFAC–derived components and leachate level.** (a) F_max_ of C1 and leachate level, (b) F_max_ of C2 and leachate level, (c) F_max_ of C3 and leachate level, (d) DOC conc. and leachate level

# **S17 Fig. Percentages of FRI–derived components and PARAFAC–derived components.** (a) integration volume of Region I (P1) and Region II (P2) by FRI method, (b) components (F­_max_ C3, F_max_ (C1 + C2)) by EEM-PARAFAC method among ten sampling points during 13 sampling months

# **References**

1. Park M, Snyder SA. Sample handling and data processing for fluorescent excitation–emission matrix (EEM) of dissolved organic matter (DOM). Chemosphere. 2018; 193: 530–537. https://doi.org/10.1016/j.chemosphere.2017.11.069

2. Kothawala DN, Murphy KR, Stedmon CA, Weyhenmeyer GA, Tranvik LJ. Inner filter correction of dissolved organic matter fluorescence. Limnol Oceanogr: Methods. 2013; 11(12): 616–630. https://doi.org/10.4319/lom.2013.11.616

3. Lakowicz JR. (ed). Principles of fluorescence spectroscopy. Springer science & business media; 2013. <https://doi.org/10.1007/978-0-387-46312-4_2>

4. Bro R. PARFAC. Tutorial and Applications. Chemom Intell Lab Syst. 1997; 38(2): 149–171. <https://doi.org/10.1016/S0169-7439(97)00032-4>

5. Stedmon CA, Bro R. Characterizing dissolved organic matter fluorescence with parallel factor analysis: a tutorial. Limnol Oceanogr: Methods. 2008; 6(11), 572–579. https://doi.org/10.4319/lom.2008.6.572

6. Bro R, Kiers HA. A new efficient method for determining the number of components in PARAFAC models. J Chemom. 2003; 17(5), 274–286. <https://doi.org/10.1002/cem.801>

7. Murphy KR, Stedmon CA, Graeber D, Bro R. Fluorescence spectroscopy and multi-way techniques. PARAFAC. Anal Methods. 2013; 5(23): 6557–6566. <https://doi.org/10.1039/C3AY41160E>

8. Stedmon CA, Bro R. Characterizing dissolved organic matter fluorescence with parallel factor analysis: a tutorial. Limnol Oceanogr: Methods*.* 2008; 6(11), 572–579.

[https://doi.org/10.4319/lom.2008.6.572](https://doi.org/10.4319/lom.2008.6.572 9)

[9](https://doi.org/10.4319/lom.2008.6.572 9). Lorenzo-Seva U, Ten Berge JM. Tucker's congruence coefficient as a meaningful index of factor similarity. Methodology. 2006; 2(2): 57. <https://doi.org/10.1027/1614-2241.2.2.57>

10. Chen W, Westerhoff P, Leenheer JA, Booksh K. Fluorescence excitation− emission matrix regional integration to quantify spectra for dissolved organic matter. Environ Sci Technol. 2003; 37(24): 5701–5710. https://doi.org/10.1021/es034354c

11. He XS, Fan QD. Investigating the effect of landfill leachates on the characteristics of dissolved organic matter in groundwater using excitation–emission matrix fluorescence spectra coupled with fluorescence regional integration and self-organizing map. Environ Sci Pollut Res. 2016; 23: 21229–21237. https://doi.org/10.1007/s11356-016-7308-7

12. Cui Y, Wu Q, Yang M, Cui F. Three-dimensional excitation-emission matrix fluorescence spectroscopy and fractions of dissolved organic matter change in landfill leachate by biological treatment. Environmental Science and Pollution Research. 2016; 23: 793–799. http:// dx.doi.org/10.1007/s11356-015-5226-8.

13. Osburn CL, Del Vecchio R, Boyd TJ. Physicochemical effects on dissolved organic matter fluorescence in natural waters. Aquatic organic matter fluorescence; 2014. pp233–277. https://doi.org/10.1017/CBO9781139045452.012

14. Chen WB, Smith DS, Guéguen C. Influence of water chemistry and dissolved organic matter (DOM) molecular size on copper and mercury binding determined by multiresponse fluorescence quenching. Chemosphere. 2013; 92(4), 351–359. https://doi.org/10.1016/j.chemosphere.2012.12.075

15. Lee YK, Hong S, Hur J. Copper-binding properties of microplastic-derived dissolved organic matter revealed by fluorescence spectroscopy and two-dimensional correlation spectroscopy. Water Res. 2021; 190: 116775. https://doi.org/10.1016/j.watres.2020.116775

16. Hansen AM, Kraus TE, Pellerin BA, Fleck JA, Downing BD, Bergamaschi BA. Optical properties of dissolved organic matter (DOM): Effects of biological and photolytic degradation. Limnol Oceanogr. 2016; 61(3): 1015–1032. https://doi.org/10.1002/lno.10270

17. Weishaar JL, Aiken GR, Bergamaschi BA, Fram MS, Fujii R, Mopper K. Evaluation of specific ultraviolet absorbance as an indicator of the chemical composition and reactivity of dissolved organic carbon. Environ Sci Technol. 2003; 37(20): 4702–4708. https://doi.org/10.1021/es030360x

18. McKnight DM, Boyer EW, Westerhoff PK, Doran PT, Kulbe T, Andersen DT. Spectrofluorometric characterization of dissolved organic matter for indication of precursor organic material and aromaticity. Limnol Oceanogr*.* 2001; 46(1): 38–48. https://doi.org/10.4319/lo.2001.46.1.0038

19. Cory RM, McNeill K, Cotner JP, Amado A, Purcell JM, Marshall AG. Singlet oxygen in the coupled photochemical and biochemical oxidation of dissolved organic matter. Environ Sci Technol. 2010;44(10): 3683–3689. https://doi.org/10.1021/es902989y

20. Ohno T. Fluorescence inner-filtering correction for determining the humification index of dissolved organic matter. Environ Sci Technol. 2002; 36(4): 742–746. https://doi.org/10.1021/es0155276

21. Huguet A, Vacher L, Relexans S, Saubusse S, Froidefond JM, Parlanti E. Properties of fluorescent dissolved organic matter in the Gironde Estuary. Org Geochem. 2009; 40(6): 706–719. https://doi.org/10.1016/j.orggeochem.2009.03.002

22. Parlanti E, Wörz K, Geoffroy L, Lamotte M. Dissolved organic matter fluorescence spectroscopy as a tool to estimate biological activity in a coastal zone submitted to anthropogenic inputs. 2000; Org Geochem.  31(12), 1765–1781. https://doi.org/10.1016/S0146-6380(00)00124-8

23. Wilson HF, Xenopoulos MA. Effects of agricultural land use on the composition of fluvial dissolved organic matter. 2009; Nat Geosci, 2(1): 37–41. https://doi.org/10.1038/ngeo391

24. Coble PG. Characterization of marine and terrestrial DOM in seawater using excitation emission matrix spectroscopy. 1996; Mar Chem51: 325–346. https://doi.org/10.1016/0304-4203(95)00062-3

25. Helms JR, Stubbins A, Perdue EM, Green NW, Chen H, Mopper K. Photochemical bleaching of oceanic dissolved organic matter and its effect on absorption spectral slope and fluorescence. Mar Chem. 2013; 155, 81–91. https://doi.org/10.1016/j.marchem.2013.05.015

26. Baker A, Bolton L, Newson M, Spencer RG. Spectrophotometric properties of surface water dissolved organic matter in an afforested upland peat catchment. Hydrol Process. 2008; 22(13), 2325–2336. https://doi.org/10.1002/hyp.6827

27. Murphy KR, Stedmon CA, Wenig P, Bro R. OpenFluor–an online spectral library of auto-fluorescence by organic compounds in the environment. Anal Methods. 2014; 6(3): 658–661. https://[doi.org/10.1039/C3AY41935E](https://doi.org/10.1039/C3AY41935E)
